# Improving Nonviral Gene Delivery by Activating Mechanosensing-Dependent Endocytic Pathways

**DOI:** 10.1021/acsami.5c23767

**Published:** 2026-01-08

**Authors:** Flaminia Fruzzetti, Beatrice Ruzzante, Eleonora Giagnorio, Silvia Bonanno, Giuseppe Lauria Pinter, Stefania Marcuzzo, Gabriele Candiani, Nina Bono

**Affiliations:** † genT_LΛB, Dept. of Chemistry, Materials and Chemical Engineering 18981 “Giulio Natta”, Politecnico di Milano, Via Bassini 6, Milan 20133, Italy; ‡ Neurology 4- Neuroimmunology and Neuromuscular Diseases Fondazione 9328 IRCCS Istituto Neurologico “Carlo Besta”, Via Celoria 11, Milan 20133, Italy; § ALS Centre, Third Neurology Unit, Fondazione IRCCS Istituto Neurologico “Carlo Besta”, Via Celoria 11, Milan 20133, Italy; ∥ Department of Medical Biotechnology and Translational Medicine, University of Milan, Milan 20133, Italy; ⊥ Brain-Targeted Nanotechnologies (BraiNs) Lab Fondazione, IRCCS Istituto Neurologico “Carlo Besta” − Politecnico di Milano, Via Celoria 11, Milan 20133, Italy

**Keywords:** non-viral gene delivery, mechanotransduction, transfection, mechanical stimulation, gene transfer techniques, YAP, endocytosis

## Abstract

The delivery of nucleic acids into host cells has emerged as an innovative and promising therapeutic approach for various diseases. Despite significant advances in nanoparticle delivery systems, persistent cellular barriers limit the clinical application of most existing technologies. In this study, we developed a programmable device that applies precise uniaxial cyclic stretching to cells cultured on custom polydimethylsiloxane chambers to investigate whether mechanical stimulation can enhance the transfection efficiency (TE) of gold-standard non-viral gene delivery vectors. Applying cyclic mechanical stimulation (*f* = 0.1 Hz, ε = 10% strain, *t* = 30 min) to HeLa cells and human myoblasts (hMyo) significantly increased nuclear translocation of the mechanosensitive transcription factor Yes-Associated Protein (YAP). Gene expression analysis revealed that this mechanical conditioning orchestrated a coordinated modulation of endocytic machinery, upregulating clathrin-mediated endocytosis (FCHO1) and macropinocytosis (STX1B) pathways while downregulating endocytic inhibitors (DLC1, EHD2). These mechanically induced cellular adaptations resulted in significantly enhanced TE of both plasmid DNA (pDNA)- and mRNA (mRNA)-carrying gold-standard branched polyethylenimine (*b*PEI)-based complexes in both HeLa cells and hMyo, compared to static conditions. Our findings demonstrate that mechanical stimulation is an effective complementary strategy for improving non-viral gene delivery by leveraging endogenous cellular mechanotransduction pathways. Rather than modifying vector chemistry, this mechanobiological approach enhances the performance of existing delivery systems by transiently modulating cellular uptake capacity and nuclear accessibility. This work offers mechanistic insights into how mechanotransduction regulates cellular uptake and highlights opportunities for leveraging controlled mechanical environments in applications such as ex vivo cell engineering.

## Graphical abstract

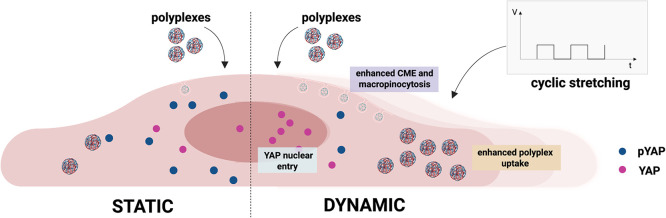

## Introduction

Over the past decades, the delivery of nucleic acids into host cells has emerged as an innovative therapeutic approach to treat, cure, or prevent diseases.^1^ Accordingly, gene delivery techniques have become essential in molecular medicine, offering promising treatment options for cancer, inherited pathological conditions, and viral infections, including recent applications in COVID-19 vaccines.^2^ Despite being the safest approach, the delivery of naked genetic material remains rather inefficient because of two fundamental challenges: nucleic acids’ anionic nature at physiological pH prevents spontaneous crossing of similarly charged cell membranes, and once in the extracellular environment, nucleic acids face rapid degradation by ubiquitous nucleases.^3,4^

To overcome these limitations, research has focused on different approaches, which may be broadly classified into physical methods and vector-based strategies.^5^ Physical gene delivery strategies employ external forces to enhance the intracellular delivery of nucleic acids into the cytosol or cell nucleus,^6–9^ while vectors protect nucleic acids from degradative enzymes and promote complete delivery pathway, from internalization into target cells through endosomal escape, cytoplasmic trafficking, nuclear entry (for DNA only), to cargo release.^10^ Though viral vectors demonstrate high innate transduction efficiency, their application is limited by tropism, immunogenicity, and toxicity concerns. Consequently, safer and less immunogenic non-viral vectors have gained increasing attention, despite their limited transfection efficacy.^11^

Non-viral vectors typically utilize cationic lipids or polymers that spontaneously self-assemble with anionic nucleic acids to form nano- and microparticles (lipoplexes and polyplexes, respectively). These complexes possess physicochemical properties enabling cell membrane crossing through either passive fusion (particularly for lipoplexes) or active endocytosis mechanisms, including clathrin- and caveolae-mediated pathways.^12–15^

Despite significant advances in non-viral gene delivery systems, cellular barriers limit their clinical application.^4,16–19^ In recent decades, nanomedicine and gene delivery research have primarily focused on engineering the physicochemical properties of nanoparticles/complexes—such as size, shape, and surface chemistry—to enhance cellular interactions and cargo delivery.^20,21^ However, these conventional strategies often encounter persistent challenges with transfection efficiency (TE), cytotoxicity, cellular uptake, and intracellular trafficking, suggesting that a fundamentally different approach is needed.

Recent studies have revealed that mechanical forces play a crucial role in cellular trafficking and endocytosis, opening unexplored opportunities for enhancing gene delivery.^22–25^ It has been shown that mechanical stimulation may induce changes in membrane tension,^26^ cytoskeletal organization,^27^ and intracellular signaling pathways^28^ that potentially facilitate genetic material uptake and processing. In this light, the role of intracellular molecular pathways in bionano interactions has often been overlooked. Cells naturally respond to mechanical cues in their microenvironment, suggesting that leveraging these native mechanosensitive pathways could provide a more physiologically relevant approach to gene delivery as a promising area for modulating nanoparticle uptake.

Here, we investigate how fundamental mechanobiology principles can be strategically harnessed to overcome non-viral gene delivery limitations. We designed a programmable cell stretching device to integrate controlled mechanical stimulation with non-viral transfection protocols and enhance non-viral vectors’ performance through activating mechanosensitive cellular pathways. Our experimental strategy was designed to investigate mechanotransduction-mediated modulation of cellular uptake. We employed branched polyethylenimine (*b*PEI), a widely used non-viral vector whose uptake occurs primarily through active endocytic pathways, making it well suited for dissecting mechanically regulated endocytosis.^29^ We next examined how cyclic mechanical stretching influences DNA- and mRNA-polyplex delivery and probed the underlying mechanisms, including endocytic pathway modulation and activation of the mechanosensitive transcriptional regulator Yes-Associated Protein (YAP).^30^

Together, these analyses assess whether mechanotransduction can be harnessed to overcome cell-intrinsic barriers to non-viral gene delivery.

## Results and Discussion

### Development and Validation of a Programmable Cell Stretching Device

We designed a programmable cell culture device capable of delivering precise cyclic strain stimulation to adherent cells grown on polydimethylsiloxane (PDMS) chambers. The device integrates an Arduino-controlled stepper motor with a ball-screw mechanism to generate controlled mechanical deformation of the culture units (Figure 1A). This configuration enables the precise application of uniaxial strain along the *x*-axis, offering adjustable mechanical stimulation parameters for investigating mechanotransduction effects on cellular behavior.

**Figure 1:**
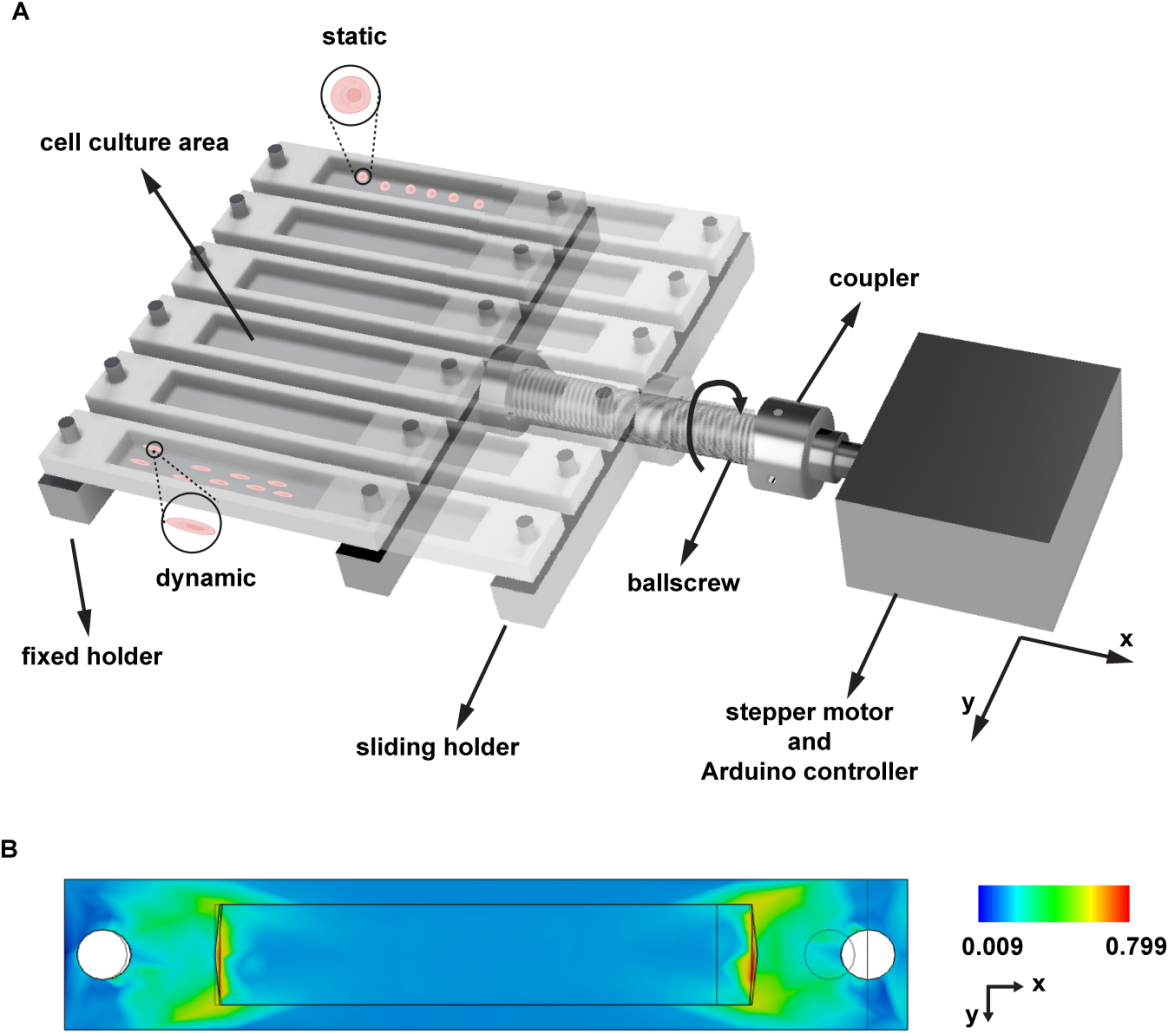
Programmable stretching device. (A) Rendering of the device setup showing mechanical stretching applied on PDMS chambers and the electro-mechanical components comprising: (i) a sliding and a fixed holder to allow culture chambers stretching, (ii) a ball-screw with a nut, (iii) a coupling element, and (iv) a stepper motor. (B) FEM simulation showing strain distribution on the PDMS culture chamber under an 8 mm deformation. The color scale represents strain values (ε_*xx*_), with the chamber oriented according to the coordinate axes shown. The simulation model was discretized into 4,829 triangular elements with a 10% average element size and one degree of freedom.

To evaluate the mechanical performance of the system, we conducted both computational and experimental analyses. Finite Element Method (FEM) simulations assessed stress distribution across the PDMS culture chambers during stretching. As illustrated in Figure 1B, strain values along the *x*-direction (ε_*xx*_) progressed from 0% at rest to ≈15% strain under maximum deformation. Using the Fusion 360 probing tool, we confirmed uniform strain distribution throughout the cell culture area, ensuring consistent mechanical stimulation to all cultured cells.

We further validated the relationship between the ball-screw rotation and the corresponding strain applied to the PDMS chamber through experimental measurements (Figure S1). Reference points within the cell culture area were tracked using ImageJ software during incremental displacement, and these measured strain values were compared with FEM predictions. Figure S2 shows excellent agreement between experimental measurements and FEM predictions, confirming the accuracy of our computational model in characterizing the mechanical behavior of the system.

Our analysis verified that a displacement of 8 mm, corresponding to one complete rotation of the ball screw, produced 15% strain in the PDMS chamber. Notably, the strain–displacement relationship exhibited nonlinear behavior, consistent with the elastomeric properties of PDMS.

The Arduino microcontroller interface enables precise programming of stimulation protocols with controlled strain parameters, as confirmed by the agreement between predicted and experimental measurements. This system enables the investigation of cellular responses across a relevant range of strain amplitudes (ε = 0–20%) and frequencies (*f* = 0–2 Hz). This programmable approach allows further systematic exploration of how mechanical forces influence key cellular processes involved in non-viral transfection, including membrane dynamics, endocytic pathway activation, and intracellular trafficking of gene delivery vectors.

### Evaluation of Mechanical Stretching on Cellular Behavior

When developing a platform for cell culture applications, it is crucial to assess the compatibility of the system with cell cultures and to determine how various stimulation parameters may affect cell viability. We first characterized the behavior of two different cell types seeded on PDMS chambers compared to traditional polystyrene (PS) culture dishes. This comparison is essential because extensive scientific evidence demonstrates that cellular behaviors, such as proliferation and gene expression, are significantly influenced by the extracellular environment. Indeed, substrates with different stiffness levels producing distinct effects on cells.^31^ Our assessment revealed no statistically significant differences in cell viability over a two-day culture period for both HeLa (Figure 2A) and human myoblast (hMyo) cells (Figure 2B) when cultured on PS vs PDMS (*p* > 0.05). Importantly, this similarity also extended to cellular proliferation rate (Figure 2C–D). This thorough validation confirmed the compatibility of the substrate and enabled us to proceed with further experiments using deformable PDMS chambers as culture substrates for mechanical stimulation studies.

**Figure 2:**
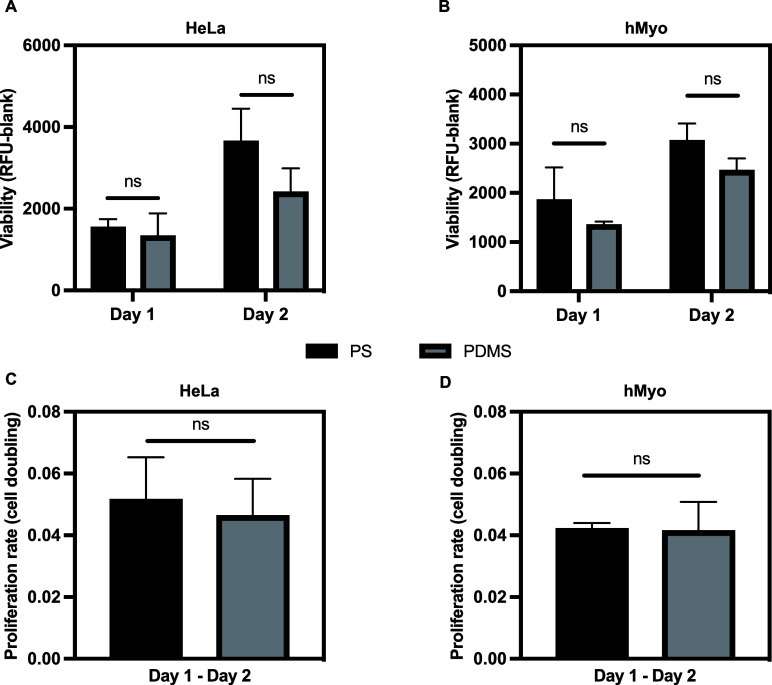
Behavior of cells cultured onto PS plate vs. PDMS culture chamber. Viability (A, B) and proliferation rate (C, D) of HeLa cells and hMyo seeded at 2 × 10^4^ cells/cm^2^ on different culture vessels. Data are expressed as mean ± SD (*n* ≥ 3). No statistically significant differences were detected between groups (ns).

Next, we systematically evaluated the effects of various cyclic mechanical stimulation regimes on cell viability. The programmable nature of our platform enabled independent modulation of both strain magnitude and frequency to identify optimal stimulation parameters that maintain cells alive while potentially enhancing their biological functions. We subjected HeLa and hMyo cells to a comprehensive experimental matrix combining three strain magnitudes (ε = 5%, 10%, and 15%) and three frequencies (*f* = 0.1, 0.5, and 1 Hz). Cell viability was assessed immediately after stimulation. As illustrated in Figure 3, both cell types exhibited clear strain- and frequency-dependent responses, with cell viability progressively decreasing under conditions of higher mechanical stress. Under moderate conditions (ε = 5% and 10% combined with lower *f* = 0.1 and 0.5 Hz), cells maintained a relatively high viability. However, even at these strain levels, exposure to the highest frequency (*f* = 1 Hz) caused a marked decrease in viability, particularly at 10% strain, where viability was lower than 40%. The most pronounced effects occurred at ε = 15%, where viability decreased substantially across all frequencies, with the most severe reduction observed at *f* = 1 Hz (viability <20%). Notably, at 15% strain with 0.1 and 0.5 Hz frequency, HeLa cells were still moderately viable, demonstrating that frequency is a critical determinant of cellular tolerance to mechanical stress alongside strain magnitude. This progressive decline in cell viability with increasing frequency and strain magnitude suggests that cellular adaptation mechanisms become overwhelmed during more intense mechanical stimulation, potentially compromising membrane integrity and cell–substrate adhesion. Based on these findings, we established safety parameters for all subsequent mechanotransfection experiments, constraining cell stimulation protocols to *f* ≤ 0.5 Hz (specifically 0.1 and 0.5 Hz) and ε ≤ 10% (specifically 5% and 10%). These parameters provide sufficient mechanical stimulus while maintaining cellular health necessary for successful gene delivery and expression.

**Figure 3:**
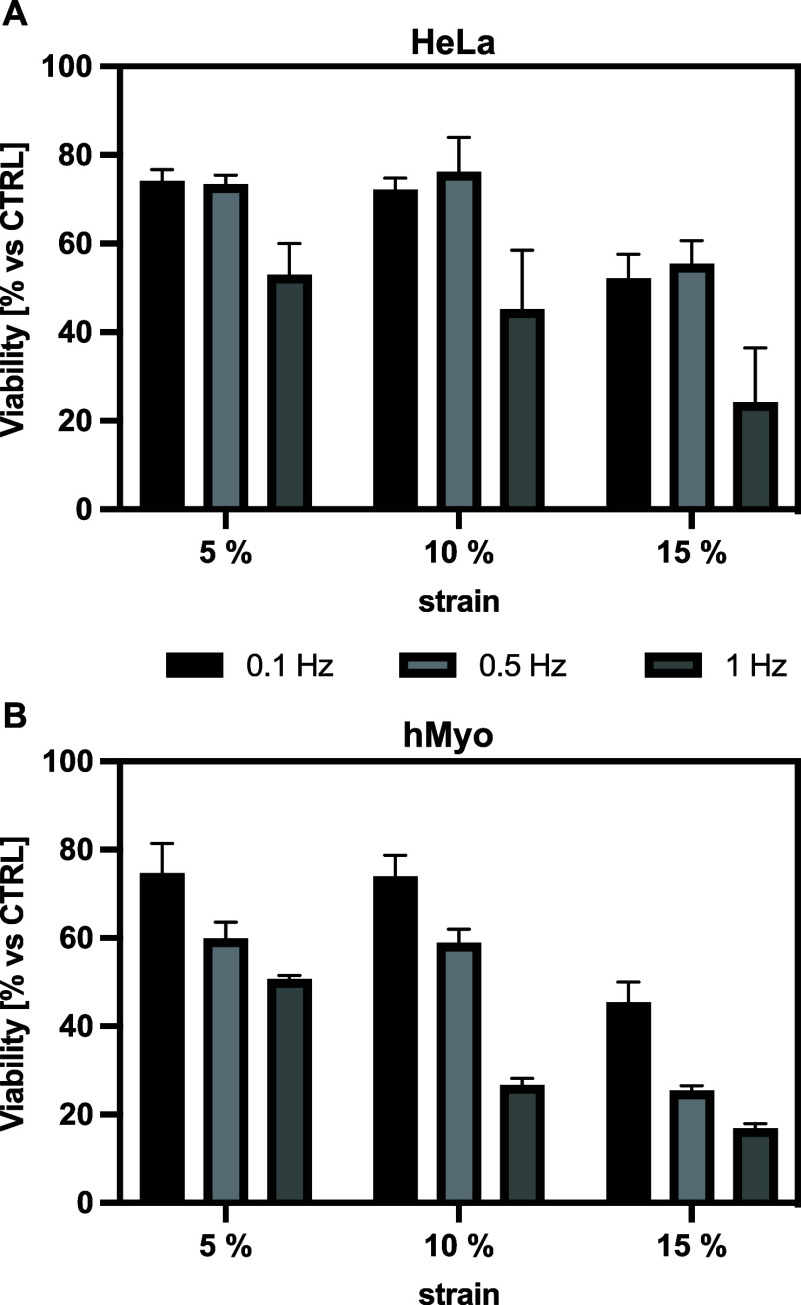
Effect of 30 min mechanical stimulation on cell viability. Quantitative assessment of cell viability (%) in (A) HeLa and (B) hMyo cells subjected to cyclic mechanical stimulation under varying strains (ε = 5%, 10%, and 15%) and frequencies (*f* = 0.1, 0.5, and 1 Hz). Stimulation was applied continuously for 30 min. Unstimulated cells cultured on identical PDMS chambers served as controls (100% viability). Results are expressed as mean ± SD (*n* ≥ 3).

### Enhanced Gene Delivery through Cyclic Mechanical Stimulation Protocols

To investigate whether controlled mechanical stimulation could enhance intracellular nucleic acid delivery and increase transgene expression, we challenged cells with nonviral gene delivery vectors under optimal mechanostimulation conditions (*f* ≤ 0.5 Hz, ε ≤ 10%) (a protocol named “mechanofection”). For this purpose, the gold-standard transfectant branched PEI (*b*PEI) was used to prepare *b*PEI/plasmid DNA (pDNA), as previously described.^18,32^

The experimental timeline of mechanotransfection is depicted in Figure 4A. Briefly, after 24 h postseeding, polyplexes were added to HeLa and hMyo cells, followed immediately by 30 min cyclic stretching at defined strain magnitudes (ε = 5% or 10%) and frequencies (*f* = 0.1 or 0.5 Hz). Transfection efficiency (TE) was assessed 24 h postmechanical stimulation and compared to unstimulated transfected controls (polyfection), where *b*PEI-based complexes were delivered to cells under conventional static conditions. Polyfection represents gold-standard *b*PEI-based delivery under conventional static conditions and serves as the positive control throughout all experiments for comparison with mechanofection.

**Figure 4:**
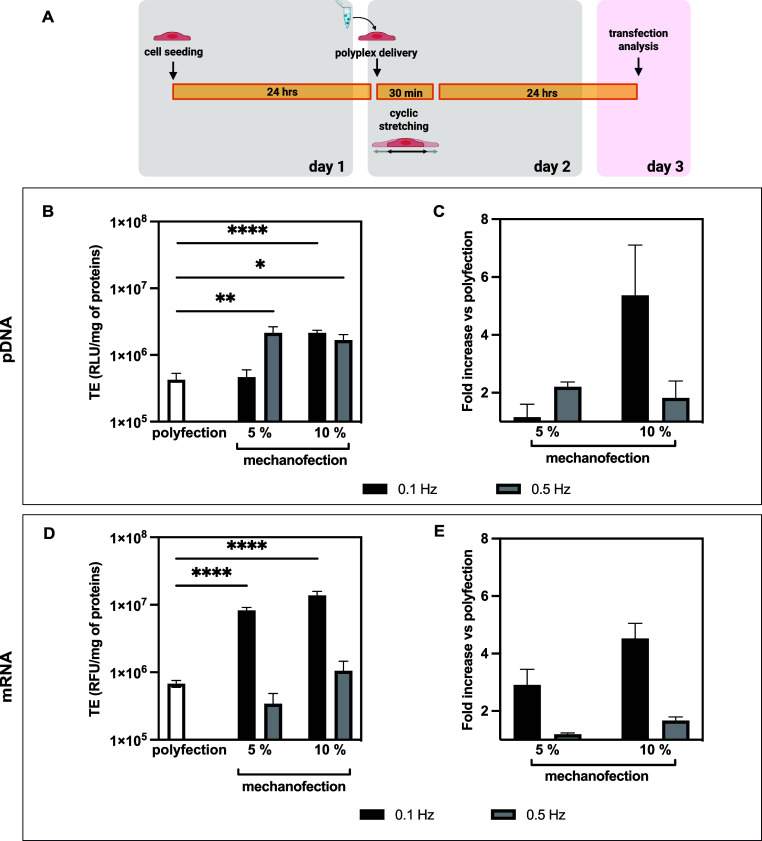
Comparative transfection efficiencies between mechanotransfection vs. polyfection. (A) Experimental workflow of the cyclic stretching. Twenty-four hrs postseeding, HeLa cells were challenged with polyplexes, stimulated for 30 min at different frequencies (*f* = 0.1 and 0.5 Hz), then cultured for an additional 24 h under standard culture conditions. Transfection efficiency (TE) was assessed 24 h after polyplex administration. We used different reporter systems appropriate for each nucleic acid type: (B) luciferase activity (RLU/mg of proteins) for DNA TE and (D) fluorescence intensity (RFU/mg of proteins) for mRNA TE. Both measurements were normalized to total protein content to account for potential variations in cell number between conditions. Polyfection served as the conventional reference method throughout all experiments and enhancement. TE is expressed as a fold-increase in transgene expression of mechanotransfected over statically transfected cells (mechanofection vs polyfection) for (C) bPEI/pDNA polyplexes, and (E) bPEI/mRNA polyplexes. Results are expressed as mean ± SD (*n* ≥ 3) (*p < 0.05; **p < 0.01; ****p < 0.0001).

Our results demonstrate that applying cyclic mechanical stretching significantly improved the delivery of DNA-containing polyplexes in both HeLa and hMyo cells compared to conventional static polyfection (*p* < 0.05). Notably, lower frequency stimulation (*f* = 0.1 Hz) combined with higher strain (ε = 10%) consistently yielded the highest enhancement across both cell types. This optimal condition can be explained by several biomechanical factors. At very low frequencies, cells have sufficient time between stretching cycles for extensive cytoskeletal reorganization and optimal activation of mechanotransduction pathways that enhance endocytosis.^33^ Furthermore, as illustrated in Figure 3, this parameter combination (*f* = 0.1 Hz, ε = 10%) maintains high cell viability (>70%) while providing sufficient mechanical stimulus to robustly activate cellular mechanosensing pathways.

Overall, stimulation at *f* = 0.1 Hz and ε = 10% yielded optimal outcomes, resulting in a significant increase in TE for both HeLa (Figure 4B) and hMyo cells (Figure S3A).

The differential response magnitude between cell lines (HeLa) and primary cells (hMyo) likely reflects their distinct mechanosensitive properties and endocytic capacities. This cell type-dependent response resulted in ≈5-fold and ≈3-fold increases in TE for HeLa (Figure 4C) and hMyo cells (Figure S3B), respectively. This fold-enhancement across different cell types highlights the generalizability of our mechanotransduction-based approach and suggests that the underlying mechanisms—modulation of endocytic pathways through mechanosensitive signaling—are broadly conserved cellular responses. Moreover, this highlights the potential of mechanical stimulation for enhancing gene delivery in primary cells, which generally exhibit lower TE than immortalized cell lines.^34^

To further confirm that mechanical stimulation enhances the efficacy of non-viral gene delivery vectors across different types of genetic cargo, we conducted additional experiments with mRNA delivery. We prepared *b*PEI/mRNA complexes and applied the same mechanical stimulation protocol as previously described. Both HeLa (Figure 4D–E) and hMyo cells (Figure S3C,D) exhibited enhanced fluorescent protein (mCherry) expression when treated with *b*PEI/mRNA polyplexes followed by cyclic 30 min stimulation, with fold-increases comparable to those observed for pDNA delivery (≈5-fold).

Importantly, the similar fold-enhancement observed for both pDNA and mRNA delivery provides critical mechanistic insight. Since mRNA translation occurs directly in the cytosol without requiring nuclear entry, while pDNA must translocate to the nucleus for transcription, the comparable enhancement for both cargo types indicates that the primary mechanism of action is enhanced cellular uptake through modulation of endocytic pathways, rather than differential effects on nuclear import or transgene transcription.

These findings align with published studies reporting the effects of cyclic stimulation on nanoparticle internalization and gene transfer across various cell types.^35^ Mechanical stretch has been shown to induce multiple biological responses, including plasma membrane remodeling, cytoskeletal alterations, activation of specific cell signaling pathways, and upregulation of transcription factors, that, in turn, regulate endocytic machinery.^36,37^ We thus hypothesized that these mechanically induced cellular adaptations collectively contribute to the enhanced TE observed in our mechanotransfection approach.

### Investigating the Temporal Pattern and Mechanisms of Stretch-Enhanced Transfection

To elucidate the mechanisms underlying the enhanced gene delivery observed with mechanical stimulation, we investigated both the temporal dynamics of stimulation and its effects on cellular endocytic pathways. This comprehensive approach allowed us to determine both the optimal timing of mechanical stimulation and its impact on cellular endocytic machinery.

First, we examined whether the timing and pattern of mechanical stimulation (cyclic vs noncyclic) differentially affect gene transfer outcomes. We conducted two distinct experimental series: one investigating the temporal relationship between cyclic stimulation and polyplex administration, and another comparing cyclic vs noncyclic (single event) stimulation patterns.

For the first set of experiments, we maintained constant frequency and strain parameters (f = 0.1 Hz and ε = 10%, respectively) while varying the sequence and duration of mechanical stimulation with respect to polyplex administration. Two distinct protocols were tested: *i)* 30 min cyclic stimulation first, followed by polyplex delivery (Cycl Stim_30 min_ + Poly Add) (Figure S4A); and *ii)* one-hr cyclic stimulation first, followed by polyplex delivery (Cycl Stim_1 h_ + Poly Add) (Figure S4B). As shown in Figure S4C, the latter two protocols (polyplex delivery after cyclic mechanical stimulation) led to a small but significant increase in TE compared to statically transfected cells. This response suggests that cellular adaptations induced by mechanical stimulation persist after the cessation of stimulation, leading to a more responsive cell behavior for subsequent transfection. This observation aligns with established literature demonstrating that mechanical stimulation influences cellular mechanisms, including membrane trafficking,^35,38–40^ proliferation,^28^ and cytoskeletal organization.^15,32,41^ To determine whether cyclic stimulation is specifically required or if single mechanical transitions could produce similar effects, we conducted a second set of experiments comparing noncyclic vs cyclic stimulation patterns. In these experiments, single-event deformation protocols (one-time stretch followed by one-time recovery) were employed. Specifically, cells were seeded and cultured for 24 h in standard conditions, and then challenged with a single dynamic stretching event, i.e., moving the chamber from ε = 0% to 10%, maintaining the strain for 30 min (f = 0 Hz), after which the chamber was moved back to the initial configuration (ε = 0%) and polyplexes were finally delivered to the cells (Figure S5A). Overall, no statistically significant differences in TE were observed (*p* > 0.05) for this noncyclic, single-event deformation protocol compared to static controls (Figure S5B). The absence of enhanced TE with single-event deformation protocols demonstrates that repeated cycles of stretching and relaxation are essential to induce the cellular adaptations that facilitate an improved TE. These findings suggest that the dynamic nature of cyclic stimulation, rather than static deformation alone, is crucial for activating the mechanotransduction pathways that enhance non-viral gene delivery. Mechanical strain stimulation may induce rapid plasma membrane rearrangements as cells actively maintain their integrity under changing tension conditions.^35,42^ This dynamic membrane remodeling process, essential for cell mechanoadaptation,^43^ involves coordinated cytoskeletal reorganization that facilitates adaptive changes in cell surface area in response to mechanical stimuli. The orchestrated cellular responses to alternating mechanical states likely contribute to the enhanced TE observed in our mechanotransfection experiments, reminiscent of mechanisms exploited by other physical methods that leverage membrane dynamics for improved delivery.^26^ Our methodical approach ensured that any observed effects on TE could be attributed specifically to mechanotransduction-related processes rather than nonspecific stress responses or cellular damage.

### Mechanical Stimulation Orchestrates the Modulation of Endocytic Pathways

Understanding the cellular internalization mechanisms of nanoparticles is critical for optimizing their design and therapeutic efficacy. There is a consensus that distinct endocytic pathways contribute to polyplex internalization.^44,45^ Currently, the major recognized uptake pathways include clathrin-mediated endocytosis (CME), fast endophilin-mediated endocytosis (FEME), clathrin-independent carrier (CLIC)/GEEC pathway, macropinocytosis, phagocytosis, and caveolae-mediated endocytosis.^46^ While the physical properties of polyplexes influence which endocytic pathways mediate their uptake, the efficiency and route of internalization can be further modulated by external stimuli. In this context, clathrins likely play a key role in rapidly compensating for sudden changes in cell membrane structure, whereas other endocytic pathways and actin remodeling require more time to activate.^36,47^ Our temporal pattern experiments demonstrated that cyclic mechanical stimulation specifically enhances TE, suggesting alterations in cellular machinery. We therefore hypothesized that mechanical forces might directly modulate endocytic pathways to create a more favorable behavior for promoting gene delivery. To test this hypothesis, we conducted a comprehensive gene expression analysis of key endocytosis pathway components in both HeLa and hMyo cells. We selectively quantified transcripts encoding critical mediators of various endocytic mechanisms, including pathway initiators, regulatory elements, and inhibitory factors that collectively govern nanoparticle internalization (reported in Figures S6 and S7 and summarized in Table S1).

The gene expression analysis revealed a coordinated response across multiple endocytic pathways that logically explains the enhanced TE observed under mechanical stimulation. As illustrated in Figure 5A, mechanical stimulation triggers a precisely orchestrated modulation of endocytic machinery through three key mechanisms. First, we observed selective upregulation of initiators of specific endocytic routes. FCHO1, a critical initiator of CME that senses and induces membrane curvature during clathrin-coated pit formation,^48^ exhibited significant upregulation under dynamic conditions in both cell types (Figure 5B) (*p* < 0.05 vs. static). The substantial increase in FCHO1 expression, ≈4-fold in HeLa cells and ≈7-fold in hMyo cells, suggests a direct enhancement of CME, likely increasing the number of nucleation sites for clathrin-coated pits and accelerating endocytic vesicle formation.

**Figure 5:**
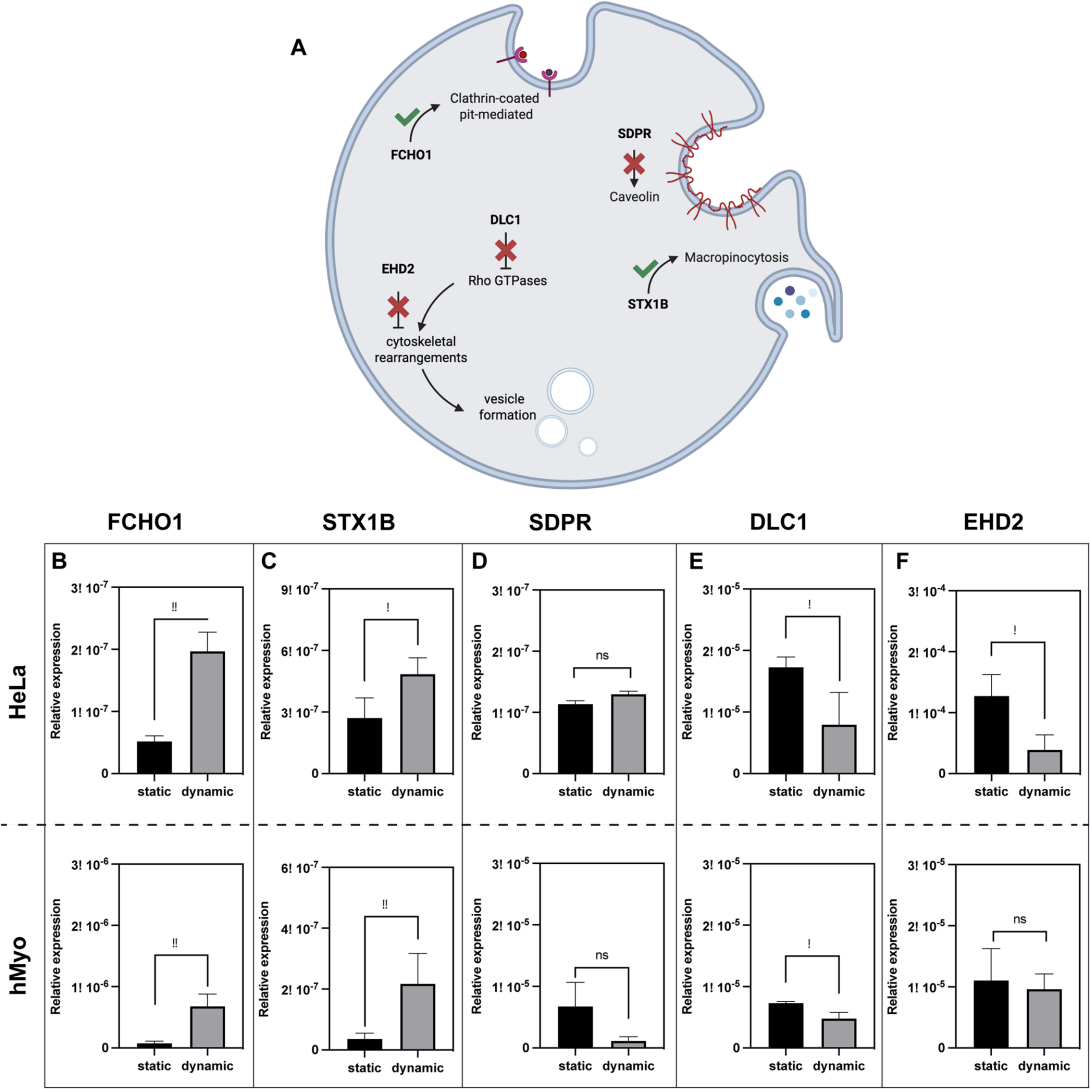
Effects of mechanical stimulation on gene expression. (A) Schematic representation of mechanically influenced transcripts: FCHO1 upregulation enhances the formation of clathrin-coated pits, DLC1 and EHD2 downregulation promotes Rho GTPases activity facilitating cytoskeletal rearrangements and vesicles formation, and STX1B upregulation enhances macropinocytosis. RT- PCR analysis of (B) FCHO1, (C) STX1B, (D) SDPR, (E) DLC1, and (F) EHD2 genes in mechanotransfected cells with respect to their polyfected counterparts. Data are means of relative expression ± SD (*n* ≥ 3) (*p < 0.05; **p < 0.01).

Second, we identified pathway-specific responses that indicate engagement of multiple internalization mechanisms. STX1B (Syntaxin 1B), recently implicated in macropinocytosis beyond its classical role in exocytosis,^49^ showed significant upregulation in both cell types under dynamic conditions (Figure 5C) (*p* < 0.05 vs. static). This finding suggests that mechanical stimulation simultaneously activates macropinocytosis, a nonselective endocytic process that allows cells to internalize large volumes of extracellular fluid through extensive membrane protrusions. In contrast, SDPR, essential for caveolae formation,^50^ showed no significant changes under mechanical stimulation conditions (Figure 5D) (*p* > 0.05 vs. static), indicating that caveolae-mediated endocytosis is not substantially affected by our stimulation.

Third, and perhaps most intriguingly, we observed concurrent downregulation of inhibitory regulators of endocytosis. DLC1, which functions as a molecular brake on endocytosis by limiting cytoskeletal reorganization through its GAP activity on Rho GTPases,^51^ showed a significant reduction in expression under mechanical stimulation (Figure 5D) (*p* < 0.05 vs. static). Similarly, EHD2, a negative regulator of membrane dynamics,^52^ was significantly downregulated in mechanically stimulated cells (Figure 5F) (*p* < 0.05 vs. static). The mechanically induced reduction in these inhibitory factors likely promotes increased Rho GTPase signaling, enhanced cytoskeletal reorganization necessary for membrane invagination, and decreased cortical tension that facilitates membrane deformation required for vesicle formation. This coordinated downregulation of inhibitory factors complements the upregulation of positive endocytic regulators such as FCHO1, creating a synergistic effect that amplifies cellular endocytic capacity.

Notably, hMyo cells showed no statistically significant differences in EHD2 expression (Figure 5F) (*p* > 0.05 vs. static), unlike HeLa cells. This molecular distinction provides a mechanistic explanation for the differential response observed between cell types, where HeLa cells exhibited a greater fold increase in TE (5-fold) compared to hMyo cells (3-fold). The persistent EHD2 expression in hMyo likely maintains a degree of membrane stabilization that partially counteracts the enhanced endocytic activity induced by mechanical stimulation, resulting in a moderated transfection enhancement compared to HeLa cells.

These coordinated transcriptional changes—upregulation of endocytic pathway initiators (FCHO1, STX1B) and downregulation of endocytic inhibitors (DLC1, EHD2)—provide molecular evidence that mechanical stimulation specifically enhances the early, rate-limiting step of cellular uptake at the plasma membrane barrier. Collectively, these findings support a mechanotransduction model wherein cyclic mechanical forces orchestrate a precisely coordinated response across the endocytic machinery, rather than influencing later intracellular barriers such as endosomal escape. This conclusion is supported by both temporal and mechanistic considerations. First, our 30 min mechanical stimulation window is well-matched to the rapid kinetics of endocytic uptake (occurring within seconds to minute), but is temporally incompatible with endosomal maturation and escape, which occur hours after internalization. By the time polyplexes undergo endosomal escape—typically >6 h post-transfection^53^—mechanical stimulation had ceased more than 5 h earlier.

Moreover, endosomal escape mediated by cationic polymers such as *b*PEI is predominantly a passive, chemically driven process through the “proton sponge effect,” involving the high buffering capacity of *b*PEI leading to osmotic swelling and membrane rupture.^54,55^ This represents an intrinsic chemical property of the ionizable polymer rather than an active cellular process that could be modulated by mechanotransduction signaling.

Overall, our results demonstrate that mechanotransduction can be harnessed as a targeted approach to improve non-viral gene delivery efficiency by specifically addressing the plasma membrane barrier, providing mechanistic insights for developing advanced transfection strategies that leverage cellular mechanosensitivity.

### Mechanical Stretching Enhances YAP Nuclear Translocation and Facilitates Transgene Transcription

Following the identification of molecular changes in endocytic pathways, we sought to elucidate the mechanotransduction pathway linking mechanical forces to these coordinated cellular responses. Specifically, we investigated whether Yes-Associated Protein (YAP), a key mechanosensitive transcription factor coactivator in the Hippo signaling pathway,^56^ serves as the critical mediator linking mechanical stimulation to the transcriptional reprogramming of endocytic genes and the enhanced gene delivery outcomes we observed. YAP activity is regulated through phosphorylation: phosphorylated state (pYAP) remains sequestered and transcriptionally inactive in the cytosol, whereas dephosphorylated YAP translocates to the nucleus to engage TEAD transcription factors and initiate gene expression.^57^ This, in turn, has been found to govern cell proliferation, cytoskeletal organization, and cellular adaptation to mechanical stimuli.^58^

Based on YAP’s established role in mechanotransduction, we hypothesized that mechanical stimulation would promote pYAP dephosphorylation and nuclear translocation, potentially contributing to the observed outcomes. To investigate this mechanism, we examined whether cells subjected to mechanical stretching would exhibit enhanced nuclear YAP accumulation compared to static controls. We conducted experiments subjecting cells to optimal cyclic mechanical stimulation (*f* = 0.1 Hz, ε = 10%, *t* = 30 min), then visualized YAP localization using immunofluorescence analyses and validated the data through Western Blot (WB). Our investigations specifically focused on the differential distribution of YAP and (cytosolic) pYAP between nuclear and perinuclear regions. Notably, our experimental setup enabled us to observe total YAP (including both pYAP and nonphosphorylated YAP) and pYAP using two different antibodies. As shown in Figure 6, CTRL cells (kept under static conditions) (Figure 6A and Figure S8A) exhibited predominantly pYAP, that is, YAP retained in its phosphorylated state, localized within the cytosol with limited nuclear presence. When exposed to mechanical stimulation, both HeLa and hMyo cells showed a marked nuclear YAP accumulation, suggesting that dephosphorylation and subsequent nuclear translocation occurred (Figure 6B and Figure S8B). This shift in localization becomes particularly evident in the merged images, where the total YAP signal (in green) shows stronger colocalization with the blue nuclear stain. These findings align with previous studies demonstrating that specific mechanical cues can trigger YAP dephosphorylation, enabling nuclear translocation and transcriptional activity.^57–60^

**Figure 6:**
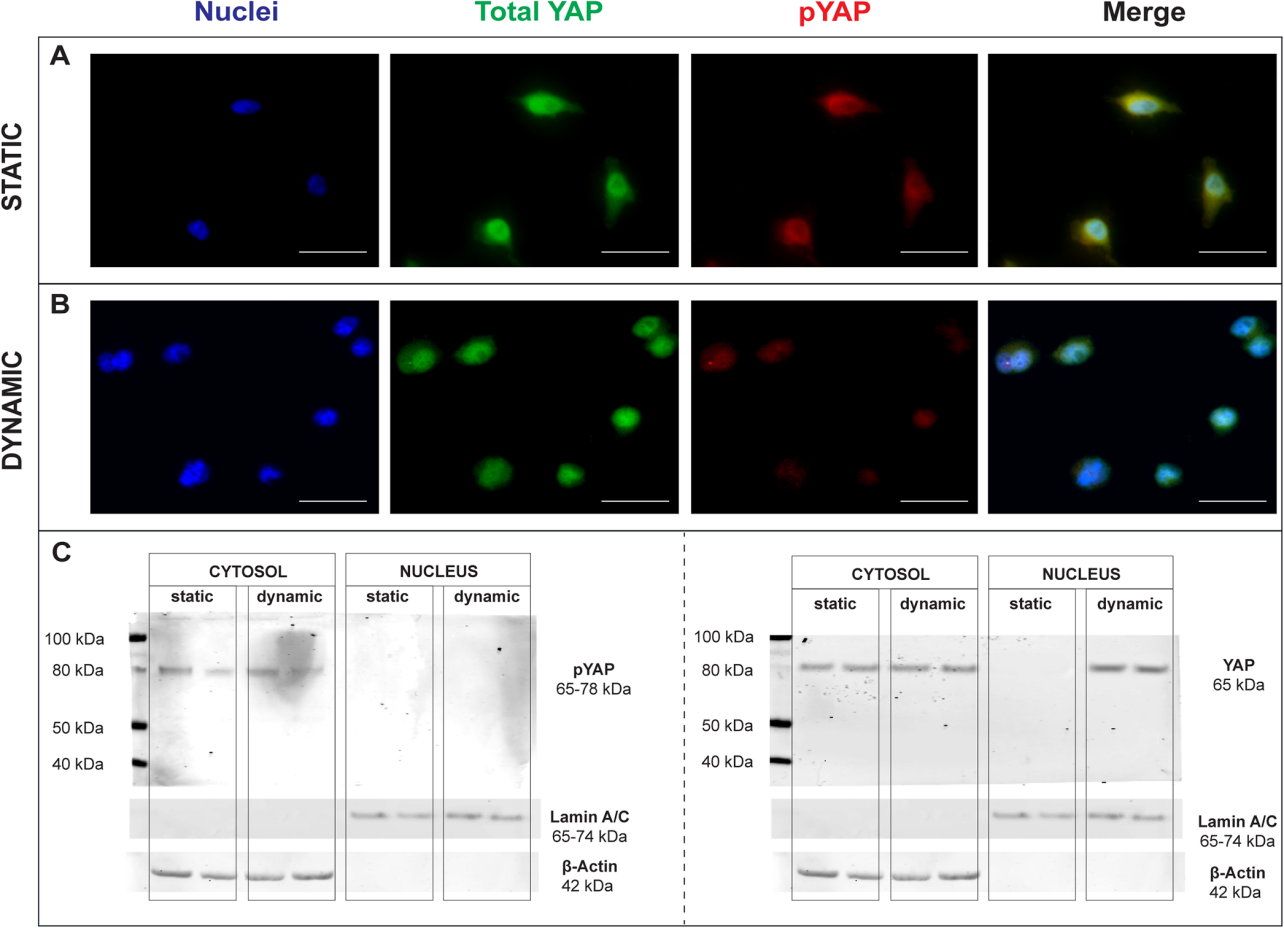
YAP localization analysis. Mechano-induced YAP nuclear translocation via immunostaining in HeLa cells (A) after 24 h in standard culture conditions vs (B) cells that underwent cyclic mechanostimulation (*f* = 0.1 Hz, ε = 10%, *t* = 30 min). Nuclei are shown in blue, while total YAP and pYAP are in green and red, respectively. Scale bars = 50 μm. (C) Mechano-induced YAP nuclear translocation via Western Blot in HeLa cells, where cytoplasmic and nuclear fractions were isolated from cells cultured under static and mechanically stimulated (dynamic) conditions. Protein extracts were probed with antibodies against total YAP (65 kDa band) and pYAP (65–78 kDa band). β-actin (42 kDa band) and Lamin A/C (65–74 kDa band) were used as the cytoplasmic loading control and the nuclear loading controls, respectively.

To corroborate these observations at the protein level, we performed WB analysis of nuclear and cytoplasmic fractions isolated from cells cultured under both static and dynamic conditions (*f* = 0.1 Hz, ε = 10%, *t* = 30 min). Results are shown in Figure 6C for HeLa and Figure S8C for hMyo. As expected, based on the established YAP regulation mechanism, phosphorylated YAP (pYAP, 65–78 kDa) was predominantly detected in the cytoplasmic fraction under static conditions, consistent with cytoplasmic sequestration of the inactive, phosphorylated form. No nuclear YAP was found in CTRL cells.

When we analyzed cells that had undergone mechanical stimulation, we observed a markedly different distribution pattern. While pYAP was still detected in the cytoplasmic fraction, total YAP exhibited pronounced enrichment in the nuclear fraction specifically upon mechanical stimulation. This shift from predominantly cytoplasmic localization under static conditions to substantial nuclear accumulation under dynamic conditions provides direct biochemical evidence that mechanical stimulation promotes YAP dephosphorylation and nuclear translocation. To assess the purity and quality of subcellular fractionation, β-actin (42 kDa) and Lamin A/C (65–74 kDa) were used as cytoplasmic and nuclear markers, respectively. Both markers were detected exclusively in their expected compartments, confirming the reliability of the fractionation procedure.

The nuclear translocation of YAP observed in our study provides a mechanistic link between mechanical stimulation and the enhanced gene delivery we observed. We thus propose a mechanotransduction model where cyclic mechanical stimulation orchestrates an integrated cellular response: YAP activation drives transcriptional reprogramming of the endocytic machinery (upregulation of FCHO1 and STX1B, downregulation of DLC1 and EHD2), creating a cellular state with enhanced capacity for polyplex internalization at the plasma membrane. This enhanced cellular uptake, which is one of the rate-limiting steps in non-viral gene delivery, explains the substantial and comparable improvements in transgene expression observed for both pDNA and mRNA polyplexes. Once internalized through this mechanically enhanced uptake phase, both cargo types undergo their respective intracellular processing through normal pathways independent of the initial mechanical stimulus: endosomal escape via *b*PEI’s intrinsic proton sponge effect, followed by mRNA translation in the cytosol or pDNA nuclear import and transcription.

Recent evidence has established YAP as a master regulator of cellular uptake mechanisms and endocytic machinery. YAP has been shown to directly regulate genes involved in CME, membrane trafficking, cytoskeletal remodeling, and vesicle formation—all processes critical for nanoparticle internalization. Upon nuclear translocation, YAP interacts with TEAD transcription factors to modulate expression of endocytic pathway components, establishing YAP as a key transcriptional regulator at the interface between mechanotransduction and cellular uptake capacity.^56,61^

Overall, our work provides significant mechanistic insights into how mechanical forces differentially modulate cell membrane trafficking. The identification of YAP as a key mediator in mechanically enhanced gene delivery opens new avenues for optimizing nonviral transfection strategies through targeted manipulation of mechanotransduction pathways.

## Conclusions

Despite notable advances, the development of non-viral gene delivery systems continues to face significant challenges. Substantial progress has been made in optimizing vector properties, including polymer structure, surface chemistry, particle size, and targeting ligands. However, the cellular mechanisms that determine uptake efficiency and intracellular trafficking remain unclear. Clarifying how these regulatory pathways influence nanoparticle uptake is a critical knowledge gap. In this study, we examined whether and how cellular mechanotransduction pathways affect non-viral gene delivery efficiency. We focused on the YAP signaling axis, a master regulator of responses to mechanical stimuli. To explore this, we engineered a programmable uniaxial cell-stretching device to precisely control mechanical parameters, including strain magnitude, frequency, and stimulation duration. This platform enabled us to identify specific biomechanical conditions (*f* = 0.1 Hz, ε = 10%, *t* = 30 min) that significantly enhance TE while maintaining high cell viability. Using gold-standard *bP*EI-based polyplexes as a model delivery system, we observed substantial improvements in gene delivery for both plasmid DNA and mRNA compared with conventional static transfection.

Our mechanistic investigations revealed that this enhancement operates through mechano-induced transcriptional regulation of endocytic machinery. Gene expression analysis demonstrated that cyclic mechanical stretching orchestrates the upregulation of key endocytic initiators (FCHO1) and macropinocytosis mediators (STX1B) while concurrently downregulating inhibitory regulators (DLC1, EHD2). This coordinated molecular response establishes an optimal intracellular microenvironment for polyplex internalization. Simultaneously, we observed that mechanical stimulation activates the YAP mechanotransduction pathway, as evidenced by dephosphorylation of pYAP and nuclear translocation of YAP, potentially providing a direct mechanistic link between our applied mechanical forces and the observed transcriptional changes in endocytic machinery.

In summary, this work establishes that cellular mechanotransduction represents a fundamental regulatory mechanism controlling cellular uptake capacity for gene delivery vectors. Our mechanotransduction-based approach enhances the critical early barrier of cellular uptake, allowing subsequent intracellular trafficking and escape to proceed through the intrinsic properties of the delivery vector.

Future investigations will aim to deepen understanding of the mechanotransduction-endocytosis axis and its broader implications. Several key questions merit further exploration. First, identifying whether additional mechanosensitive pathways beyond YAP contribute to the regulation of endocytic machinery and elucidating how these pathways interact to coordinate cellular uptake capacity will provide a more complete picture of mechanotransduction-regulated uptake. Second, determining whether mechanical conditioning affects cellular barriers beyond uptake—such as endosomal escape or nuclear import—through mechanisms not captured in our current study remains an important area for investigation.

In perspective, understanding how mechanical forces influence gene delivery mechanisms could inform the development of improved transfection protocols, particularly for *ex vivo* cell engineering and tissue engineering applications where controlled mechanical environments are already employed.

## Materials and Methods

### Materials

HeLa cells (human ovarian carcinoma epithelial cells, CCL-2) were purchased from the American Type Culture Collection. Human myoblasts were provided by Neuromuscular Disease Biobank (NeuMD-Besta) at Fondazione IRCCS Istituto Neurologico “Carlo Besta” (Milan, Italy). All samples obtained from the biobank had written informed consent using a form approved by the local Ethics Committee. Research was conducted according to protocols approved by the Carlo Besta institutional review board.

Twenty-five kDa *b*PEI (cat. no. 40872-7) was from Merck Life Science (Milan, Italy), while pGL3 (pDNA encoding the modified Firefy luciferase, pGL3-Control Vector, 5.256 kbp) and Luciferase Assay System were obtained from Promega (Milan, Italy). Sylgard 184 was purchased by Farnell (Milano, Italy).

Bipolar stepper motor NEMA 23, Digital Stepper Driver DM556, Arduino UNO Rev3, ball screw with nut LMK8UU, coupler 8 mm to 8 mm, Plexiglas 5 mm were from Amazon. Bicinchoninic acid (BCA) Protein Assay Kit, TRIzol Reagent, SuperScript IV VILO Kit, SuperScript VILO cDNA Synthesis Kit, TaqMan Gene Expression assays, Hoechst 33342 were from Thermo Fisher Scientific (Waltham, Massachusetts, USA). Mouse antitotal YAP ((63.7):sc-101199; cat. no. sc101199; RRID: AB_1131430) monoclonal antibody was from Santa-Cruz Biotechnology (Dallas, USA), and rabbit anti-pYAP (Ser127), cat. no 4911; RRID: AB_2218913) polyclonal antibody was from Cell Signaling Technology (Danvers, USA).

All the other reagents were from Merck Life Science unless otherwise specified.

### Design and Development of the Programmable Stretching Platform

The programmable stretching system consists of two primary components: (i) a computer-controlled mechanical actuator that applies uniaxial stretch to (ii) flexible PDMS elastomer cell culture chambers (Figure 1A). The mechanical actuation system converts rotary motion into linear displacement using a stepper motor connected to an 8 mm-ball screw. The culture chamber holders are positioned on this assembly, with one holder fixed in place while the other is connected to the ball screw via a bearing. This configuration allows the rotating screw to transmit linear motion to the movable holder, thereby stretching the PDMS chambers secured above it. The culture chambers are firmly attached to the holders using screws and bolts.

The system’s precise movement control was enabled by an Arduino UNO Rev3 microcontroller, which coordinates all electrical components in the circuit. A separate DM556 motor driver controller regulates the stepper motor’s speed and direction, configured at 200 pulses/revolution with a motor input current of 1.8 A. The Arduino code modulated motor speed, revolution time, and direction inversion intervals according to stimulation amplitude and frequency. The program employs two equivalent cycles that control clockwise and counterclockwise rotation of the screw. Direction changes of the motor were executed by setting the direction pin to either LOW (clockwise) or HIGH (counterclockwise) values. Each cycle generated precisely timed pulses that drive the motor steps, with motor speed controlled by the delayMicroseconds function, which determines the timing between activation (digitalWrite­(stepPin, HIGH)) and deactivation (digitalWrite­(stepPin, LOW)) of the motor, thus enabling precise motor movement and ultimately the stretching parameters. For the Arduino code, please refer to the (Supporting Information S3 Arduino Code section).

PDMS cell culture chambers were fabricated using a 10:1 base-to-curing agent ratio according to the manufacturer’s instructions,^62^ yielding a nominal 1 MPa stiffness. Based on FEM, we fabricated chambers with an internal seeding area of 5 cm^2^ featuring a 0.5 mm thickness and 3.5 mm height, while the overall dimensions of the culture chambers measure 80 mm in length and 15 mm in width (Figure 1A). Custom molds for these chambers were designed with Fusion 360 software and fabricated in poly methyl-methacrylate (PMMA). The final PDMS culture chambers were obtained via replica molding, followed by thorough washing with deionized water (dH_2_O) and sterilization by autoclaving.

### In Vitro Cell Culture

Mycoplasma-free HeLa cells were cultured in Dulbecco’s Modified Eagle’s Medium (DMEM) supplemented with 10% (v/v) fetal bovine serum (FBS), 1 mM sodium pyruvate, 10 mM HEPES, 100 U/mL penicillin, 0.1 mg/mL streptomycin, and 2 mM glutamine.

Mycoplasma free primary hMyo were isolated from patients’ biopsy by culturing in Dulbecco’s modified Eagle’s medium (DMEM) containing 20% heat-inactivated FBS (Thermo Fisher Scientific), 1% penicillin–streptomycin, l-glutamine, 10 μg/mL insulin (Sigma-Aldrich, St. Louis, MO), 2.5 ng/mL basic fibroblast growth factor (bFGF) (Thermo Fisher Scientific), and 10 ng/mL epidermal growth factor (EGF) (Thermo Fisher Scientific). The cells were cultured at 37 °C in a humidified atmosphere under a constant supply of 5% (v/v) CO_2_ (hereafter referred to as standard culture conditions). The medium was changed twice a week. Upon reaching 70% confluence, the cells were enzymatically dissociated using trypsin-EDTA (Sigma) and either reseeded for immediate propagation or cryopreserved in medium containing 10% (v/v) DMSO (Sigma) for further experiments.

### Evaluation of Cell Viability in Static vs Dynamic Culture Conditions

To assess the effect of cyclic strain on cell viability, HeLa and hMyo cells were seeded onto PDMS chambers at a density of 2 × 10^4^ cells/cm^2^ and maintained in standard culture conditions for 24 h.

Afterward, cells were stimulated for 30 min at (i) different cyclic strains, namely ε = 5%, 10%, and 15%, in correspondence of fixed frequencies (0.1 and 0.5 Hz), or (ii) different frequencies, namely *f* = 0.1, 0.5, and 1 Hz, in correspondence of fixed strains (ε = 5%, 10%, and 15%), then maintained in standard culture conditions. Twenty-four hours after the discontinuation of the stimulation, cell viability was evaluated using the Alamar Blue assay according to the manufacturer’s instructions. Briefly, the medium was removed from each well and replaced with 1.5 mL/chamber of 1× resazurin dye solution in cell culture medium. Next, cells were incubated in standard culture conditions for 2 h, then the fluorescence was read with a Synergy H1 reader (BioTek, Winooski, VT, USA) (λ_ex_ = 540 nm, λ_em_ = 595 nm). The viability of unstimulated cells (CTRL) was assigned to 100%, and the viability of stimulated ones was determined according to eq 1:

1 $$\mathrm{Viability}\left[\%\right]=\left[\frac{F_{\mathrm{stimulated}\;\mathrm{cells}}}{F_{\mathrm{CTRL}}}\right]\times 100$$

where *F* is the recorded fluorescence.

### In Vitro Cell Transfection Assays

Transfection assays were performed on HeLa cells and hMyo. Briefly, cells were seeded at a density of 2 × 10^4^ cells/cm^2^ on PDMS chambers and maintained in standard culture conditions for 24 h. The following day polyplexes were prepared: 25 kDa *b*PEI was diluted in 10 mM HEPES to reach a final polymeric concentration of 0.86 mg/mL and, considering that there is one nitrogen per repeating *b*PEI unit (−NHCH_2_CH_2_–, Mw = 43 Da), such concentration corresponds to an amine concentration [N] of 20 mM.^63^ After a prewarming at room temperature (r.t.) before use, polyplexes were prepared by adding the 0.25 μg/μL aqueous solution of pDNA (0.25 μg/cm^2^) or the 0.1 μg/μL aqueous solution of mRNA (0.32 μg/cm^2^) to *b*PEI solutions to give a final DNA concentration of 20 ng/μL and N/P 30, where N/P is defined as the number of amines (N) of the *b*PEI used to complex the phosphate groups (P) of a given amount of nucleic acids, and a final mRNA concentration of 10 ng/μL and N/P 20. Afterward, polyplexes were incubated for 20 min at r.t. and later administered to cells in culture chambers.

Twenty-four hours after the addition of polyplexes to the cells, cytotoxicity was evaluated by performing the Alamar Blue viability assay, as previously explained, and calculated according to eq 2:

2 $$\mathrm{Cytotoxicity}\left[\%\right]=100-\mathrm{viability}\left[\%\right]=\left(1-\frac{F_{\mathrm{transfected}\;\mathrm{cells}}}{F_{\mathrm{CTRL}}}\right)\times 100$$

Transfection effectiveness was evaluated 24 h after delivering *b*PEI/pDNA polyplex by measuring the luciferase activity in cell lysates (intracellular firefly luciferase) or the culture media (secreted *Gaussia* luciferase), depending on the pDNA used, i.e., pGL3 and pNLuc, respectively. These pDNAs contain different promoters, particularly effective for cell lines (SV40)^64^ and primary cells (CMV), respectively.^65^

To evaluate the overall luciferase activity, 20 μL of either cell lysates (for pGL3-transfected cells, HeLa) or cell supernatant (for pNLuc-transfected cells, hMyo) was mixed with 50 μL of the corresponding luciferase assay substrate. The luminescence signal (expressed as Relative Light Units, RLU) was measured using a Sinergy H1 reader.

When pGL3 was used, firefly luciferase signals were normalized to the total protein content determined by BCA assay, and the TE was expressed as RLU/mg of proteins, while when pNLuc was employed, the TE was simply related to the *Gaussia* luciferase activity expressed as RLU/well.

When mRNA, i.e., mCherry, was used, TE was evaluated 24 h after transfection by measuring fluorescence signal (expressed as Relative Fluorescence Units, RFU) in 20 μL of cell lysates using a Sinergy H1 reader. Signals were normalized to the total protein content determined by BCA assay, and the TE was expressed as RFU/mg of proteins.

### In Vitro Transfection Assays under Mechanical Stimulation

To investigate potential effects within transfection assays, the custom-made stretching device was used to stimulate cells and dynamically challenge them with *b*PEI-based polyplexes under the combinations of the stimulation parameters, which were not detrimental to cells.

Cells were seeded on culture chambers at a seeding density of 2 × 10^4^ cells/cm^2^. Following 24 h incubation under standard conditions, cells were challenged with polyplexes and mechanically stimulated. Following an additional 24-h incubation in standard conditions, an Alamar Blue assay was carried out to test cell viability, and TE was evaluated by means of pGL3 and pNLuc assays for HeLa and hMyo, respectively, and mCherry for both cell types.

To evaluate the effects of the stimulation on TE, cells were subjected to the stretching stimulus for 30 min at combinations of different frequencies (0.1 and 0.5 Hz) and strains (5% and 10%). Unstimulated cells, i.e., cells cultured under static transfection conditions, were used as internal references (hereafter referred to as static controls).

### RNA Extraction

Total RNA was extracted from 1 × 10^5^ cells. Briefly, cells were detached and resuspended in their culture medium, and 1 mL of TRIzol reagent (Thermos Fisher Scientific) was added to each cell vial. After a 5 min incubation at r.t., 0.2 mL of chloroform was added. The samples were centrifuged at 12,000 × *g* for 15 min at 4 °C. After that, the RNA-containing aqueous phase was carefully transferred into a new tube. Subsequently, 0.5 mL of isopropanol was added, and the tube was vortexed for 10 s, followed by a 10 min incubation at r.t. and centrifugation at 12,000 × *g* for 10 min. After removing the supernatant, the RNA pellet was washed with 80% ethanol, vortexed for 10 s, and centrifuged at 7,500 × g for 5 min. The supernatant was removed, and the RNA pellet was air-dried before being resuspended in RNase-free water. RNA samples were stored at −80 °C.

Extracted RNA concentration was assessed using a NanoDrop spectrophotometer (Thermo Fisher Scientific).

### cDNA Synthesis and RT-PCR for Gene Expression Analysis

Complementary DNA (cDNA) was synthesized from RNA using the SuperScript IV VILO kit according to the manufacturer’s protocol. For each reverse transcription reaction, the following components were assembled: 4 μL of SuperScript IV VILO Reaction Mix, 2 μL of SuperScript IV Enzyme, and 250 ng of RNA, with nuclease-free water added to reach a final solution volume of 20 μL.

The thermal cycling conditions were set as follows: 16 °C for 30 min for the reverse transcription step, followed by 42 °C for 30 min to ensure complete cDNA synthesis. A final incubation at 85 °C for 5 min was performed to inactivate the reverse transcriptase enzyme. Following synthesis, cDNA was stored at −20 °C.

Consequently, cDNA (10 ng) was amplified in duplicate by RT-PCR, with TaqMan Fast Advanced Master Mix and specific TaqMan gene expression assays primers for CME- (FCHO1, DNAJ, SH3GL3), caveolae-mediated endocytosis- (KNCMA1, BVES, MYOF, ATP1B1, DLC1, SPRED, EHD2), and macropinocytosis- (RAB34, PYCARD, STX1B, SDPR) related genes on the ViiA7 Real Time PCR system (Thermo Fisher Scientific). 18S rRNA was used as an endogenous control.

The thermal protocol began with an initial denaturation step at 95 °C for 20 s. This was followed by 40 amplification cycles, with each cycle consisting of 95 °C for 1 s for denaturation and 60 °C for 20 s for combined annealing and extension. Fluorescence data were collected at the end of each extension phase.

Transcriptional levels of target genes were expressed as relative values normalized against 18S rRNA levels, according to the following formula $2^{-\Delta C_{t}}$ .

### Immunofluorescence Staining

Before the fixation, the cells were washed with PBS for three times and then were fixed using 4% paraformaldehyde (Sigma) in PBS pH 7.4 for 15 min at r.t. Permeabilization and blocking were performed using 0.25% Triton X-100 (Carlo Erba Reagents, Milan, Italy) and 10% Normal Goat Serum (Thermo Fisher Scientific) in PBS for 1 h at RT. Next, cells were incubated overnight at 4 °C with the mouse antitotal YAP antibody (1:500) and rabbit anti-pYAP (1:500). Immunopositivity was revealed with Alexa Fluor 488-conjugated goat ant-rabbit IgG (1:500) and Cy3-labeled polyclonal antirabbit (1:500). Cell nuclei were counterstained with Hoechst 33342 (1:1,000).

Immunostained samples were mounted on glass slides and Images of the stained samples were then captured using an Olympus BX51WI microscope equipped with an Olympus U-LH100HG fluorescence lamp. Postprocessing and image analysis were performed using ImageJ software.

### Western Blot

Cells were detached from the culture substrate by washing with PBS followed by incubation with trypsin for 5 min at 37 °C. The cell suspension was collected by centrifugation, and the resulting pellet was washed once with PBS and stored dry at −80 °C until use. For subcellular fractioning, frozen pellets were resuspended in Hypotonic Lysis Buffer (HLB; 10 mM Tris·Cl, pH 7.5, 10 mM NaCl, 3 mM MgCl_2_, 0.3% NP-40, 10% (v/v) glycerol) supplemented with protease and phosphatase inhibitor cocktails, and incubated on ice for 10 min. The suspension was centrifuged for 2 min at 200 × *g* to separate the cytoplasmic fraction. The remaining nuclear pellet was washed once with HLB, gently resuspended, and centrifuged again at 200 × *g* for 2 min. The supernatant was removed completely, and the nuclear pellet was resuspended in Nuclear Lysis Buffer (NLB; 20 mM Tris·Cl, pH 7.5, 150 mM KCl, 3 mM MgCl_2_, 0.3% NP-40, 10% glycerol) containing protease and phosphatase inhibitors. Samples were briefly vortexed and sonicated for 15 s at 60% amplitude. Both cytoplasmic and nuclear fractions were centrifuged for 15 min at 20,000 × *g*, and the resulting supernatants were collected and stored at −80 °C until analysis.

Protein concentrations were determined using the BCA assay (Thermo Fisher Scientific, Waltham, MA). Lysates were mixed with NuPAGE LDS Sample Buffer and NuPAGE Reducing Agent and denatured at 70 °C for 10 min. Equal amounts of protein (30 μg) were separated on 10% Bis-Tris NuPAGE mini gels (Thermo Fisher Scientific) and transferred onto PVDF membranes using the iBlot 2 Dry Blotting System (Thermo Fisher Scientific). Membranes were blocked for 1 h at r.t. in 5% bovine serum albumin (BSA) dissolved in Tris-buffered saline containing 0.1% Tween-20 (TBS-T), and incubated overnight at 4 °C with the following primary antibodies: mouse anti-β-actin (1:1,000, Abcam, Cambridge, UK), mouse anti-Lamin A/C (1:1,000, sc-20681, Santa Cruz Biotechnology, Dallas, TX), mouse antitotal YAP (1:500), and rabbit anti-pYAP (1:500). After washing, membranes were incubated with infrared fluorescent secondary antibodies (LI-COR Biosciences, Lincoln, NE): goat antimouse IRDye 800CW (1:10,000) and goat antirabbit IRDye 680RD (1:10,000). Bands were visualized using the Odyssey Infrared Imaging System (LI-COR Biosciences).

### Statistical Analysis

Statistical analyses were performed using Prism 8 software (GraphPad Inc., La Jolla, CA, USA). All data collected from at least three independent experiments were initially analyzed using the D’Agostino and Pearson omnibus normality test. Unpaired *t* test and one-way ANOVA (multiple comparisons) with post hoc Tukey test were used to compare two or more experimental groups, respectively. Significance was retained when *p* < 0.05. Data are expressed as mean ± standard deviation (SD, *n* ≥ 3).

## Supplementary Material



## Notes

Section S1: Device validation—computational strain analysis and experimental strain measurement; Section S2: evaluation of cell viability and proliferation in static conditions on PDMS; Section S3: Arduino code; Figure S1: static and dynamic conditions of the culture substrate; Figure S2: relationship between applied displacement and resulting strain; Figure S3: comparative transfection efficiencies in mechanotransfection vs polyfection; Figure S4: mechanotransfection outcomes: influence of delivery sequence in HeLa cells; Figure S5: mechanotransfection outcomes in HeLa cells under different temporal sequences of mechanical stimulation; Figure S6: endocytosis-related gene expression under static vs dynamic conditions in HeLa cells; Figure S7: endocytosis-related gene expression under static vs dynamic conditions in hMyo cells; Figure S8: YAP localization analysis; Table S1: endocytosis-related genes­(PDF)
