# Supplementary material for: Improving Nonviral Gene Delivery by Activating Mechanosensing-Dependent Endocytic Pathways
Source: ACS Appl Mater Interfaces. 2026 Jan 8;18(4):6578–92. doi: 10.1021/acsami.5c23767 (PMC12884457; doi:10.1021/acsami.5c23767)
Supplement: Supplementary file 1 [file am5c23767_si_001.pdf]

Supporting Information

Improving Non-Viral Gene Delivery by Activating Mechanosensing-Dependent Endocytic Pathways

Flaminia Fruzzetti[¥], Beatrice Ruzzante[¥], Eleonora Giagnorio[#], Silvia Bonanno[#], Giuseppe Lauria Pinter[†,‡], Stefania Marcuzzo[#,§], Gabriele Candiani[¥,§], and Nina Bono[¥*]

[¥] genT_LΛB, Dept. of Chemistry, Materials and Chemical Engineering "Giulio Natta", Politecnico di Milano, Via Bassini 6, Milan 20133, Italy

[#] Neurology 4- Neuroimmunology and Neuromuscular Diseases, Fondazione IRCCS Istituto Neurologico "Carlo Besta", Via Celoria 11, Milan 20133, Italy

[†] ALS Centre, 3rd Neurology Unit, Fondazione IRCCS Istituto Neurologico "Carlo Besta", Via Celoria 11, Milan 20133, Italy

[‡] Department of Medical Biotechnology and Translational Medicine, University of Milan, Milan 20133, Italy

[§] Brain-targeted Nanotechnologies (BraiNs) Lab, Fondazione IRCCS Istituto Neurologico "Carlo Besta" – Politecnico di Milano, Via Celoria 11, Milan 20133, Italy

*** Corresponding Author**

Nina Bono

e-mail: nina.bono@polimi.it

Table of contents

SUPPORTING MATERIALS AND METHODS 3

S1. DEVICE VALIDATION 3

S1.1 COMPUTATIONAL STRAIN ANALYSIS. 3

S1.2 EXPERIMENTAL STRAIN MEASUREMENT. 3

S2. EVALUATION OF CELL VIABILITY AND PROLIFERATION IN STATIC CONDITIONS ON PDMS 3

S3. ARDUINO CODE. 4

SUPPORTING FIGURES 6

FIGURE S1: STATIC AND DYNAMIC CONDITIONS OF THE CULTURE SUBSTRATE. 6

FIGURE S2: RELATIONSHIP BETWEEN APPLIED DISPLACEMENT AND RESULTING STRAIN. 6

FIGURE S3: COMPARATIVE TRANSFECTION EFFICIENCIES IN MECHANOTRANSFECTION *VS.* POLYFECTION 8

FIGURE S4: MECHANOTRANSFECTION OUTCOMES: INFLUENCE OF DELIVERY SEQUENCE IN HELA CELLS. 9

FIGURE S5: MECHANOTRANSFECTION OUTCOMES IN HELA CELLS UNDER DIFFERENT TEMPORAL SEQUENCES OF MECHANICAL STIMULATION. 10

FIGURE S6: ENDOCYTOSIS-RELATED GENE EXPRESSION UNDER STATIC *VS.* DYNAMIC CONDITIONS IN HELA CELLS. 10

FIGURE S7: ENDOCYTOSIS-RELATED GENE EXPRESSION UNDER STATIC *VS.* DYNAMIC CONDITIONS IN HMYO CELLS. 12

FIGURE S8: YAP LOCALIZATION ANALYSIS. 13

TABLE S1: ENDOCYTOSIS-RELATED GENES. 14

REFERENCES 16

SUPPORTING MATERIALS AND METHODS

S1. Device Validation.

S1.1 Computational Strain Analysis. Finite Element Method (FEM) simulations were performed using Fusion 360® software to predict stress and strain distributions across the PDMS chambers before fabrication. The culture chamber was modeled as a deformable body composed of Sylgard 184™ silicone rubber, using mechanical properties (E = 1 MPa) obtained from in-house mechanical testing (data not shown). The model was discretized into 4,829 triangular elements. Boundary conditions were defined by fixing one end of the chamber along all three axes and applying an 8-mm displacement to the opposite end, corresponding to 10 % strain.

S1.2 Experimental Strain Measurement. Experimental measurements were conducted to validate the FEM predictions and define the relationship between ball-screw rotation and membrane strain. Three chambers of each type were subjected to incremental ball-screw rotations corresponding to 0-8 mm horizontal displacements (1 mm-steps). Two black reference marks were applied onto each chamber in a regular pattern. Digital images of the top surface were acquired at each displacement value and analyzed using ImageJ software to quantify the distance between reference points. Strain (ε) was calculated using Eq. S1:

$$\varepsilon_i = \frac{\Delta l}{l_0} = \frac{l_i - l_0}{l_0} \text{(Eq. S1)}$$

where l_i is the measured distance s at displacement i, and l_0 is the initial distance at 0 mm displacement.

S2. Evaluation of Cell Viability and Proliferation in Static Conditions on PDMS.

To assess whether cell proliferation on PDMS was comparable to that on PS, HeLa cells and human myoblasts (hMyo) were seeded at the densities of 1×10^4, 2×10^4, and 4×10^4 cells/cm^2. At days 1, 2, and 3, proliferation was assessed using the Alamar Blue® assay. The medium was replaced with 1.5 mL/well of a 10 % resazurin solution (0.2 mg/mL in cDMEM), and cells were incubated for 2 hrs under standard conditions. Fluorescence (λ_{ex} = 540 nm; λ_{em} = 595 nm) was measured with a Synergy H1 microplate reader (BioTek). Proliferation was quantified in terms of doubling time using Eq. S2:

$$\text{Doubling time} = \frac{1\ [\text{h}]}{\text{Cell Doubling}} \text{(Eq. S2)}$$

where Cell Doubling is defined in Eq. S3:

$$\text{Cell Doubling} = \frac{\log_2 \frac{\text{RFU}_{t_2}}{\text{RFU}_{t_1}}}{t_2 - t_1} \text{(Eq. S3)}$$

RFU_{t1} and RFU_{t2} correspond to fluorescence readings obtained on days 2 and 1, and days 3 and 2, respectively. Because assays were performed daily at the same time, $t_2 - t_1$ was assumed to be 24 hrs.

S3. Arduino Code. An Arduino control program was implemented to regulate the rotational motion of the stepper motor used to drive mechanical stimulation. Output pins were initialized to LOW and subsequently toggled during the loop function to generate the required pattern.

```
void setup() {
int STEP;
pinMode(8, OUTPUT);
pinMode(9, OUTPUT);
digitalWrite(8, LOW);
digitalWrite(9, LOW);
}

void loop() {
    delay (1000);
    digitalWrite(8, LOW);
    digitalWrite(9, LOW);
    for (int TURNS = 1; TURNS <= 1; TURNS++) {
    for (int STEP = 0; STEP <= 1600; STEP++) {
    digitalWrite(9, HIGH);
    delayMicroseconds(1500);
    digitalWrite(9, LOW);
    }
    }
    delay(1000);
```

```
        for (int TURNS = 1; TURNS <= 1; TURNS++) {
        digitalWrite(8, HIGH);
        for (int STEP = 0; STEP <= 1600; STEP++) {
        digitalWrite(9, HIGH);
        delayMicroseconds(1500);
        digitalWrite(9, LOW);
        }
        }
        delay(1000);
    }
```

SUPPORTING FIGURES

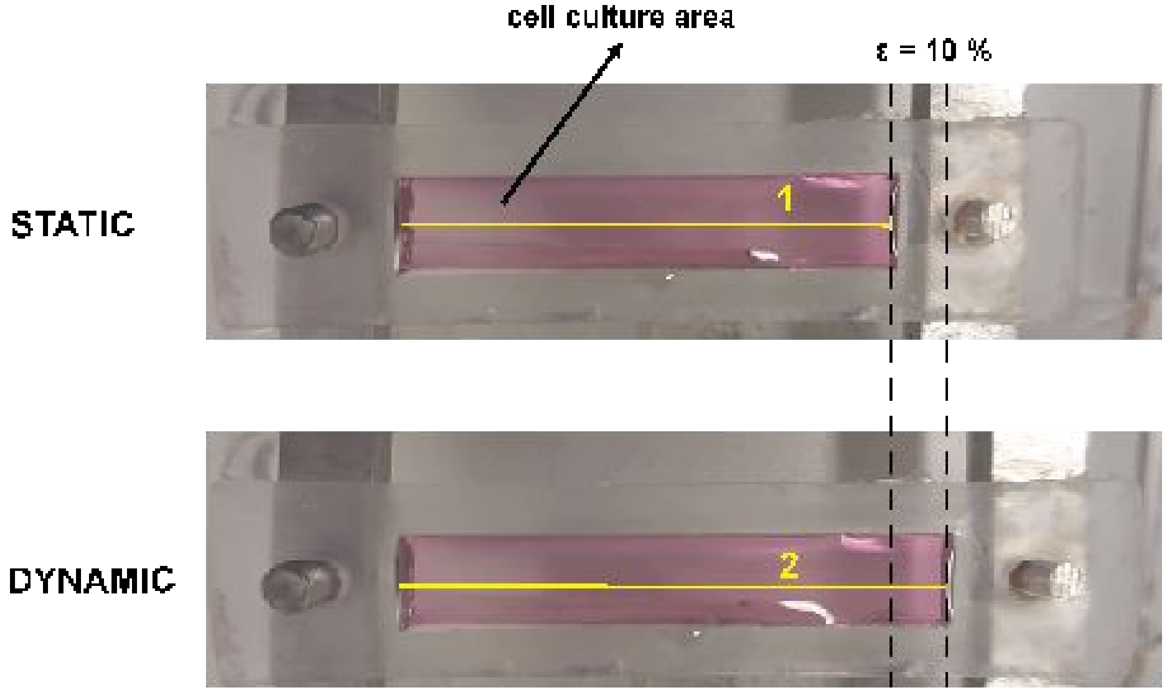

	Area	Angle	Length
1	0.037	0.000	5.000
2	0.040	0.000	5.478

$$\varepsilon(\%) = \frac{l_2 - l_1}{l_1} \times 100 = \frac{(5.478 - 5)\,\mathrm{cm}}{5\,\mathrm{cm}} \times 100 = 9.56\,\% \approx 10\,\%$$

Figure S1: Static and dynamic conditions of the culture substrate. Representative images of PDMS culture chambers under static (top panel, ε = 0 %) and dynamic (bottom panel, ε ≈ 10 %) conditions. The yellow lines (labeled as segments 1 and 2) represent reference measurements used for strain quantification. Strain measurements were performed using ImageJ software by tracking reference points along the stretching direction (x-axis). The table shows the measured lengths of two representative segments in both conditions. Scale information: culture area dimensions are 50 mm (length) × 10 mm (width), with a cell culture area of 5 cm².

Figure S2: Relationship between applied displacement and resulting strain. Experimentally measured strain values (black squares) and FEM predictions (empty gray circles) plotted as a function of the sliding-displacement. Both datasets were fitted with third-order non-linear polynomial models.

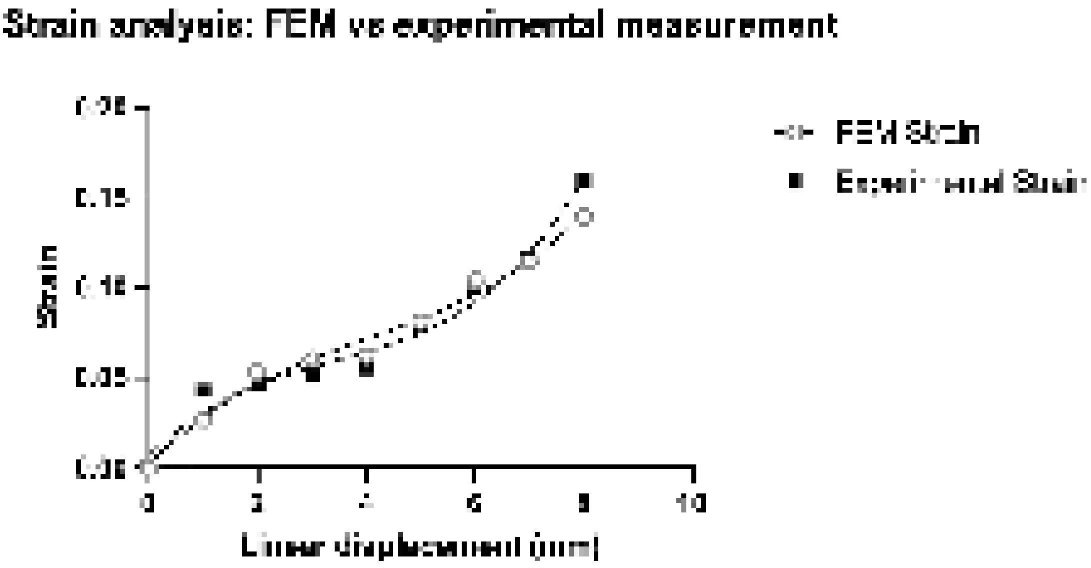

Figure S3: Comparative transfection efficiencies in mechanotransfection *vs.* polyfection. Twenty-four hrs after seeding, hMyo were exposed to polyplexes and stimulated for 30 min at 0.1 Hz or 0.5 Hz. Transfection efficiency (TE) was assessed 24 hrs later using **A)** luciferase activity (RLU/well) for DNA or **C)** fluorescence intensity (RFU/mg of proteins) for mRNA, both normalized to total protein content. Polyfection served as reference method TE is also reported as a fold-increase in transgene expression of mechanotransfected over statically transfected cells (mechanofections *vs.* polyfection) for **B)** *b*PEI/pDNA polyplexes, and **D)** *b*PEI/mRNA polyplexes. Results are expressed as mean ± SD (n ≥ 3) (*p < 0.05; **p < 0.01; ****p < 0.0001).

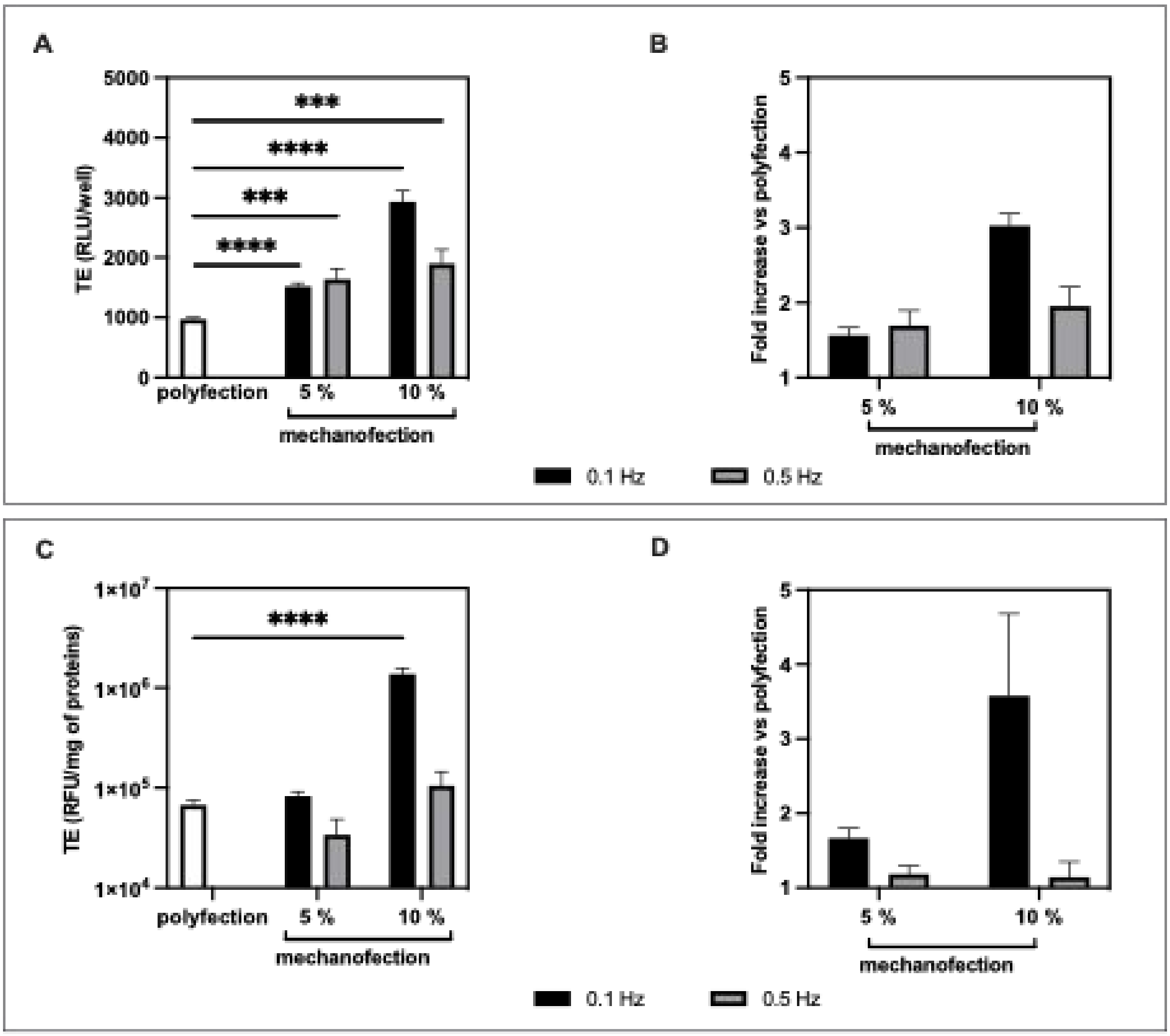

Figure S4: Mechanotransfection outcomes: influence of delivery sequence in HeLa cells. Schematic representation of mechanotransfection protocols: **A)** cells are subjected first to a 30-min cyclic stimulation (f = 0.1 Hz and ε = 10 %) and subsequently to polyplexes (Polyplex administration: Cycl Stim$_{30\ min}$ + Poly Add), and **B)** cells are subjected first to a one-hr cyclic stimulation (f = 0.1 Hz and ε = 10 %) and subsequently to polyplexes (Polyplex administration: Cycl Stim$_{1\ hr}$ + Poly Add). **C)** TE reported as RLU/mg of proteins for the abovementioned protocols.

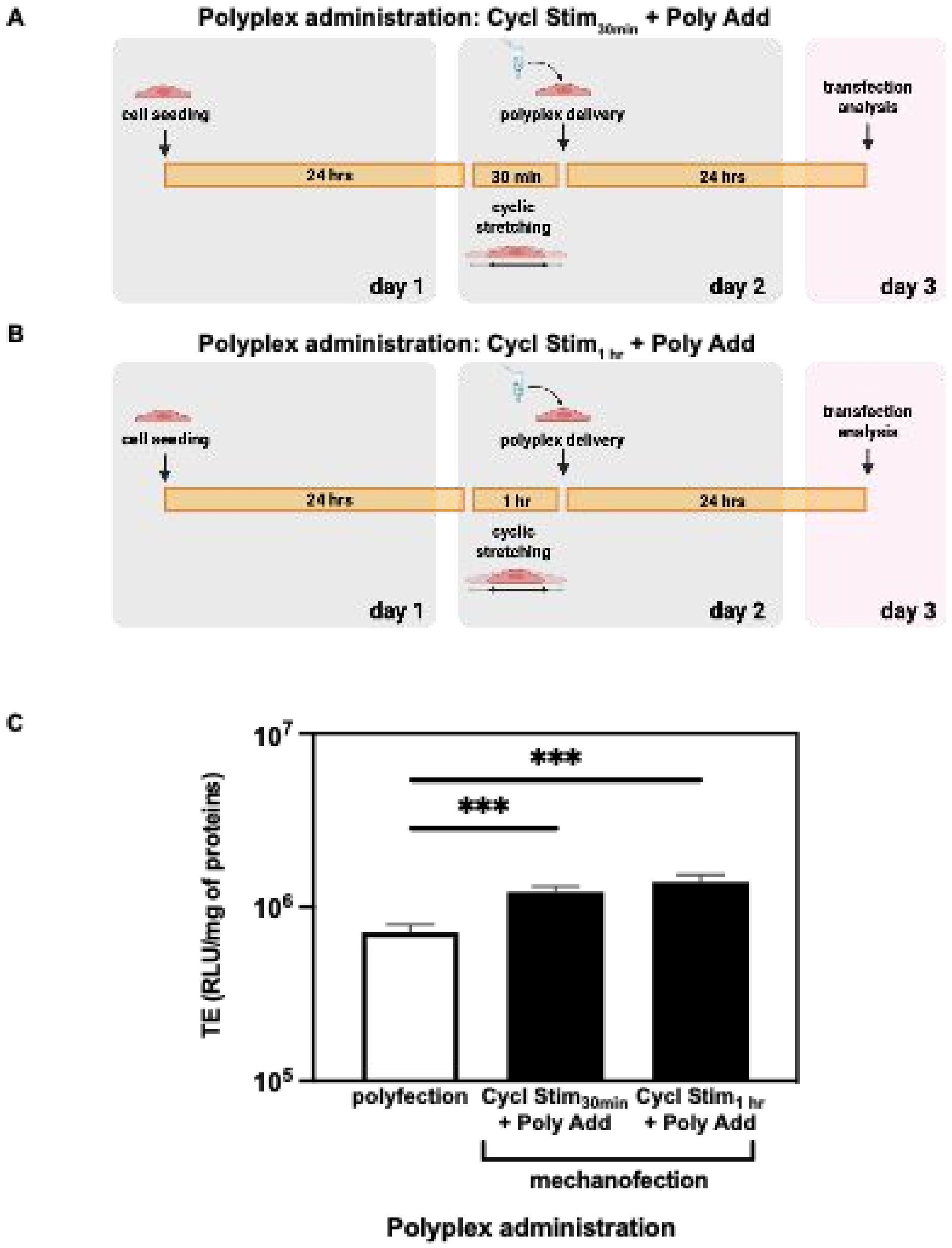

Figure S5: Mechanotransfection outcomes in HeLa cells under different temporal sequences of mechanical stimulation. A) Schematic representation of a mechanostimulation protocol where cells were seeded on a culture chamber, cultured for 24 hrs, and then challenged with a single dynamic stretching event, i.e., moving the chamber from $\varepsilon = 0$ % to 10 %, maintaining the strain for 30 min, after which the chamber was moved back to the initial configuration ($\varepsilon = 0$ %) and polyplexes were finally delivered to the cells (f = 0 Hz). **B)** TE expressed as RLU/mg of proteins compared between standard polyfection ($\varepsilon = 0$ %, f = 0 Hz), single stretching-compression stimulation ($\varepsilon = 10$ %, f = 0 Hz, t = 30 min), and cyclic stretching-compression stimulation ($\varepsilon = 10$ %, f = 0.1 Hz, t = 30 min, as reported in **Figure 4A**).

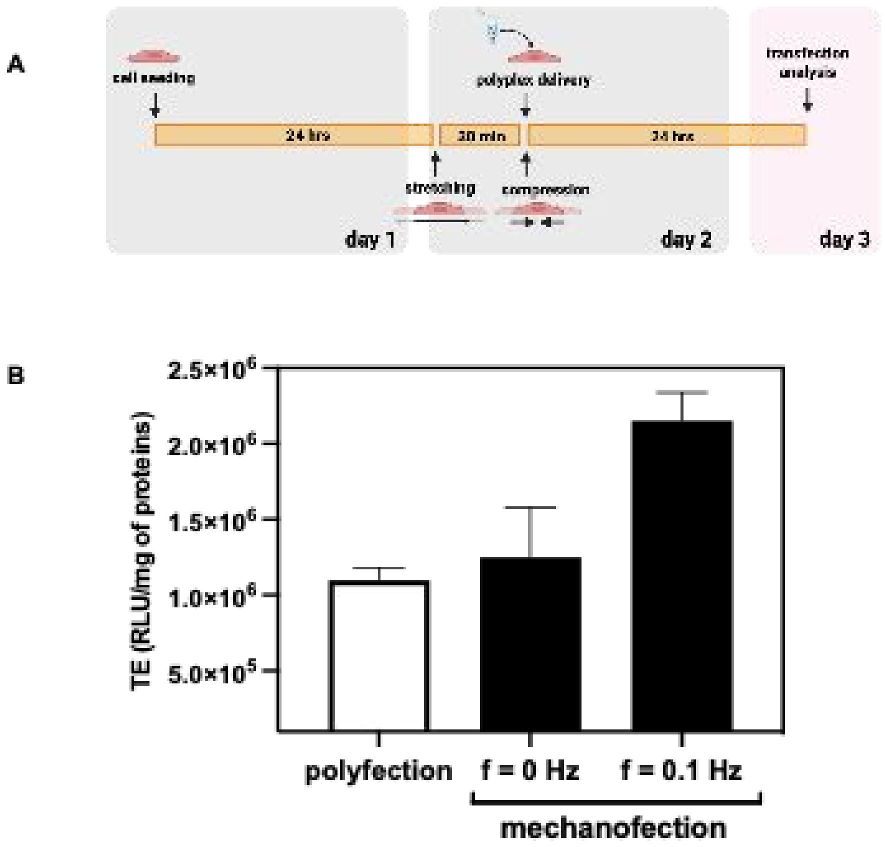

Figure S6: Endocytosis-related gene expression under static *vs.* dynamic conditions in HeLa cells. Results from rt-PCR are expressed as $2^{-\Delta C_t}$ and reported as mean ± SD for each sample group. No statistically significant differences were detected between groups.

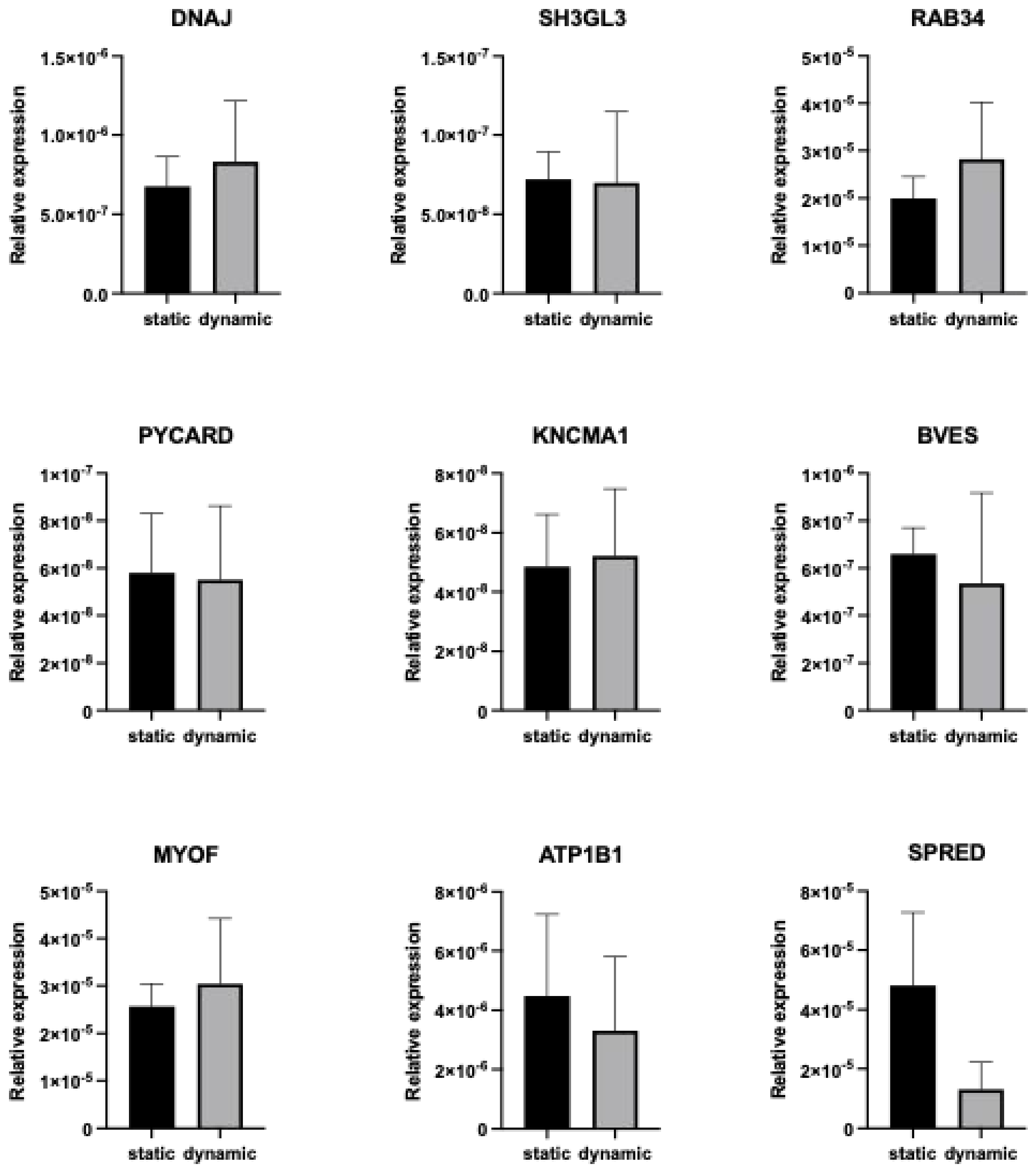

Figure S7: Endocytosis-related gene expression under static *vs.* dynamic conditions in hMyo cells. Results from rt-PCR are expressed as $2^{-\Delta C_t}$ and reported as mean ± SD for each sample group. No statistically significant differences were detected between groups.

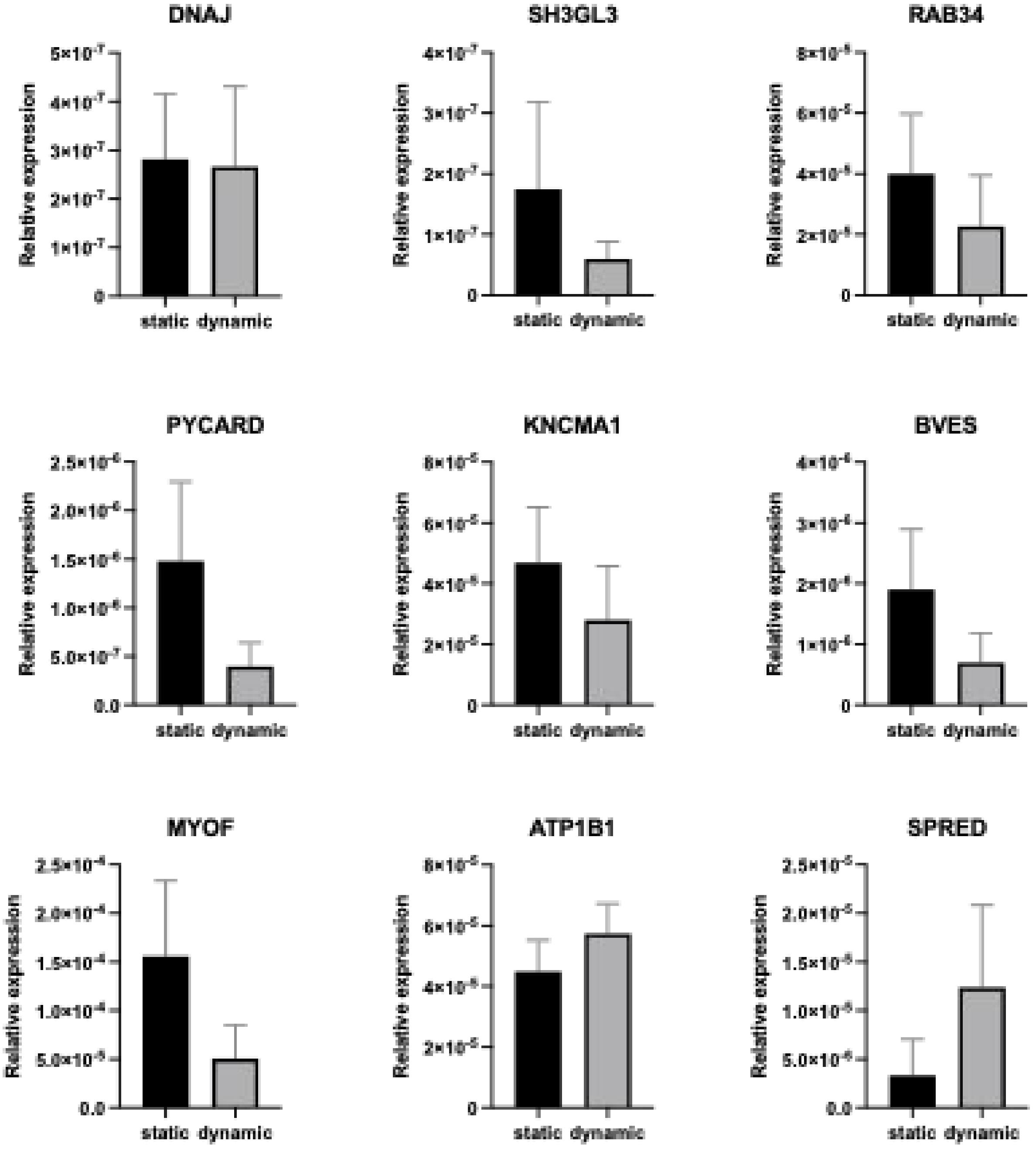

Figure S8: YAP localization analysis. Mechano-induced YAP nuclear translocation via immunostaining in hMyo cells **A)** after 24 hrs in standard culture conditions vs. **B)** cells that underwent cyclic mechanostimulation (f = 0.1 Hz, ε = 10 %, t = 30 min). Nuclei are shown in blue, while total YAP and pYAP are in green and red, respectively. Scale bars = 50 µm. **C)** Mechano-induced YAP nuclear translocation via Western Blot in hMyo cells, where cytoplasmic and nuclear fractions were isolated from cells cultured under static and mechanically stimulated (dynamic) conditions. Protein extracts were probed with antibodies against total YAP (65 kDa band) and pYAP (65-78 kDa band). β-actin (42 kDa band) and Lamin A/C (65-74 kDa band) were used as the cytoplasmic loading control and the nuclear loading controls, respectively.

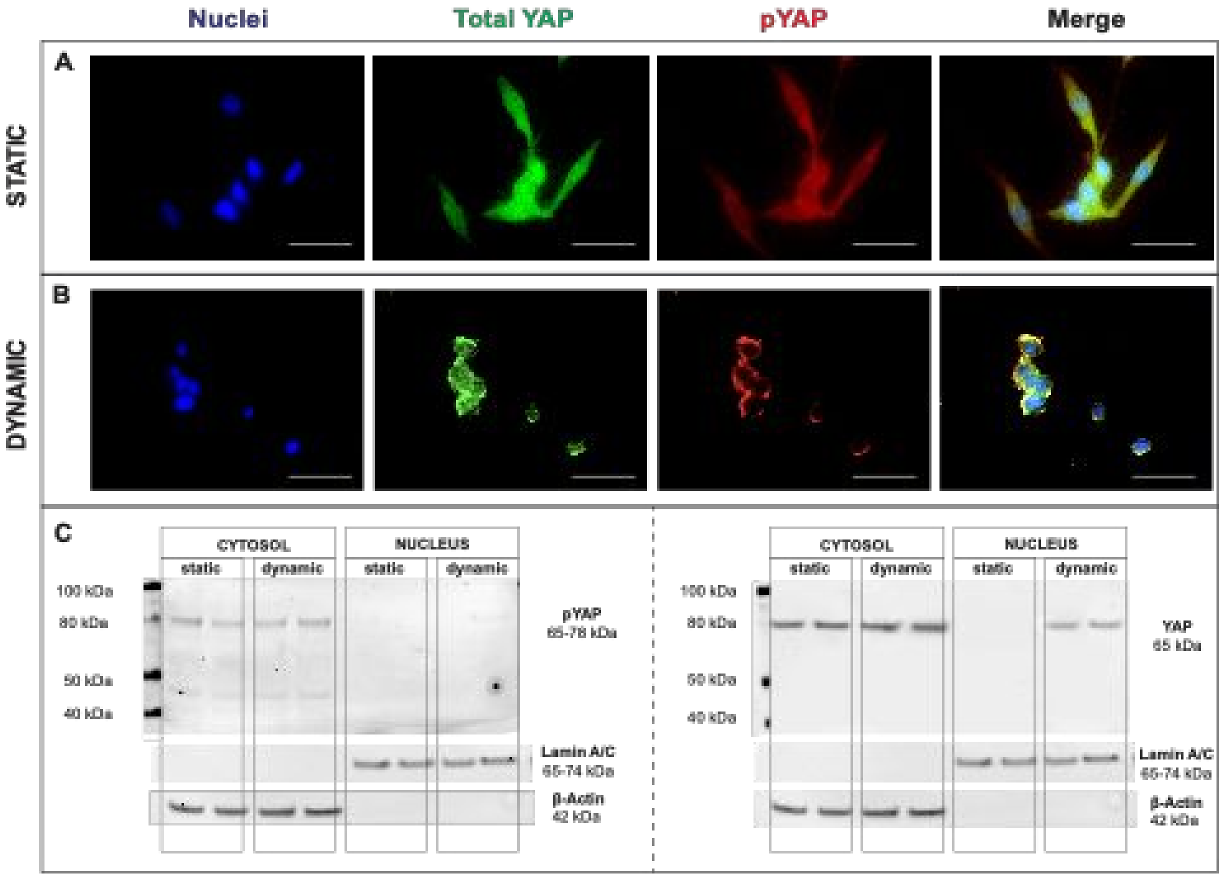

Table S1: Endocytosis-related genes.

Transcript	Function	Reference
FCHO1	Key player in clathrin-mediated endocytosis (CME); crucial factor involved in initiating the coat assembly process on the cell surface during early stages, functioning as a significant coat nucleator within clathrin-mediated processes.	[1]
SDPR	Overexpression of SDPR induces deformation of caveolae and extensive tubulation of the plasma membrane; SDPR has a direct role in the formation of caveolar invaginations; overexpressed SDPR can bind to the plasma membrane and induce membrane tubulation, implying that its role within caveolae is directly related to generating curvature.	[2]
STX1B	At the plasma membrane, STX1 and other SNAREs are not randomly distributed, but are clustered in discrete sub-micrometer-sized membrane domains that have well-understood roles in vesicle tethering, defining the sites of secretion, and regulating membrane fusion; endocytic trafficking of STX1 might also be facilitated by its localization in membrane domains, as super resolution microscopy revealed clustering of STX1 in endosomal membranes; involved in macropinocytosis.	[3]
DLC1	DLC1 acts as a molecular brake on endocytosis through its GAP activity on Rho GTPases and limits cytoskeletal reorganization necessary for vesicle formation.	[4]
EHD2	Under mechanical stress, the EHD2 ATPase is rapidly released from caveolae and translocated to the nucleus, where it regulates the transcription of several genes including those coding for caveolae constituents; EHD2 is required to maintain the caveolae reservoir at the plasma membrane during the variations of membrane tension induced by mechanical stress; it has a critical role in inhibiting endocytosis by stabilizing membrane structures and limiting membrane dynamics necessary for internalization.	[5,6]
DNAJ	Encoding proteins involved in post-endocytic recycling of vesicles.	[7]
SH3GL3	Role in clathrin-coated pit neck constriction of CME, through the BAR domain, by recruitment of both dynamin and synaptojanin, via the SH3 domain, assisting in the uncoating of the pits; A3 form participate in Fast Endophilin-Mediated Endocytosis (FEME), a dynamin-dependent, clathrin-independent endocytosis (CIE) process regulated by endophilin.	[8]
RAB34	RAB34 is a member of the Golgi secretory pathway to the plasma membrane and to the late endosomes/lysosomes, a crucial process for the delivery of acid hydrolases to the maturing lysosome.	[9]
PYCARD	Some Nod-like receptors (NLR) have been shown to regulate IL-1gamma and IL-18 secretion. NLR proteins associate with the adaptor molecule apoptotic specklike protein with a caspase-activating recruiting domain (ASC)/PYCARD, and recruit caspase-1 and caspase-5 or cardinal and caspase-1 forming a large intracellular complex coined the inflammasome; the inflammasome induces the activation of caspase-1, resulting in subsequent processing and secretion of IL-1gamma and IL-18. IL-18 plays an important role in driving the production of interferon-gamma.	[10]
KNCMA1	This gene encodes for a motif belonging to K-channels devoted to caveolin-binding.	[11]

BVES	BVES interacts with GEFT, which has previously been shown to affect cell proliferation, foci formation, neurite outgrowth, differentiation, and skeletal muscle regeneration, presumably through modulation of the Rho GTPase activity.	12
MYOF	Myoferlin is a member of the ferlin family of proteins that promotes endomembrane fusion with the plasma membrane in muscle cells and endothelial cells; myoferlin is necessary for the surface expression of vascular endothelial growth factor receptor 2 through the formation of a protein complex with dynamin-2; role for myoferlin in receptor-dependent endocytosis and an overlapping role for myoferlin-Dyn-2-Cav-1 protein complexes in membrane fusion and fission events.	13
ATP1B1	Signaling pathways related to cell adherence or cell osmoregulation. Regulation of NA^{+}/K^{+} ATP-ase.	14
SPRED	SPRED-1 is localized in cholesterol-rich membrane raft/caveola fractions and interacts with Cav-1; involved in membrane anchoring processes.	15

References

(1) Henne, W. M.; Boucrot, E.; Meinecke, M.; Evergren, E.; Vallis, Y.; Mittal, R.; McMahon, H. T. FCHo Proteins Are Nucleators of Clathrin-Mediated Endocytosis. *Science (1979)* **2010**, *328* (5983), 1281–1284. https://doi.org/10.1126/science.1188462.

(2) Hansen, C. G.; Bright, N. A.; Howard, G.; Nichols, B. J. SDPR Induces Membrane Curvature and Functions in the Formation of Caveolae. *Nat Cell Biol* **2009**, *11* (7), 807–814. https://doi.org/10.1038/ncb1887.

(3) Dingjan, I.; Linders, P. T. A.; Verboogen, D. R. J.; Revelo, N. H.; ter Beest, M.; van den Bogaart, G. Endosomal and Phagosomal SNAREs. *Physiol Rev* **2018**, *98* (3), 1465–1492. https://doi.org/10.1152/physrev.00037.2017.

(4) Kim, T. Y.; Vigil, D.; Der, C. J.; Juliano, R. L. Role of DLC-1, a Tumor Suppressor Protein with RhoGAP Activity, in Regulation of the Cytoskeleton and Cell Motility. *Cancer and Metastasis Reviews* **2009**, *28* (1–2), 77–83. https://doi.org/10.1007/s10555-008-9167-2.

(5) Torrino, S.; Shen, W.-W.; Blouin, C. M.; Mani, S. K.; Viaris de Lesegno, C.; Bost, P.; Grassart, A.; Köster, D.; Valades-Cruz, C. A.; Chambon, V.; Johannes, L.; Pierobon, P.; Soumelis, V.; Coirault, C.; Vassilopoulos, S.; Lamaze, C. EHD2 Is a Mechanotransducer Connecting Caveolae Dynamics with Gene Transcription. *Journal of Cell Biology* **2018**, *217* (12), 4092–4105. https://doi.org/10.1083/jcb.201801122.

(6) Hoernke, M.; Mohan, J.; Larsson, E.; Blomberg, J.; Kahra, D.; Westenhoff, S.; Schwieger, C.; Lundmark, R. EHD2 Restrains Dynamics of Caveolae by an ATP-Dependent, Membrane-Bound, Open Conformation. *Proceedings of the National Academy of Sciences* **2017**, *114* (22). https://doi.org/10.1073/pnas.1614066114.

(7) Ng, J.; Cortès-Saladelafont, E.; Abela, L.; Termsarasab, P.; Mankad, K.; Sudhakar, S.; Gorman, K. M.; Heales, S. J. R.; Pope, S.; Biassoni, L.; Csányi, B.; Cain, J.; Rakshi, K.; Coutts, H.; Jayawant, S.; Jefferson, R.; Hughes, D.; García-Cazorla, À.; Grozeva, D.; Raymond, F. L.; Pérez-Dueñas, B.; De Goede, C.; Pearson, T. S.; Meyer, E.; Kurian, M. A. DNAJC6 Mutations Disrupt Dopamine Homeostasis in Juvenile Parkinsonism-Dystonia. *Movement Disorders* **2020**, *35* (8), 1357–1368. https://doi.org/10.1002/mds.28063.

(8) Tim, B.; Kouznetsova, V. L.; Kesari, S.; Tsigelny, I. F. Targeting of Insulin Receptor Endocytosis as a Treatment to Insulin Resistance. *J Diabetes Complications* **2023**, *37* (11), 108615. https://doi.org/10.1016/j.jdiacomp.2023.108615.

(9) Goldenberg, N. M.; Grinstein, S.; Silverman, M. Golgi-Bound Rab34 Is a Novel Member of the Secretory Pathway. *Mol Biol Cell* **2007**, *18* (12), 4762–4771. https://doi.org/10.1091/mbc.e06-11-0991.

(10) Pedra, J. H. F.; Sutterwala, F. S.; Sukumaran, B.; Ogura, Y.; Qian, F.; Montgomery, R. R.; Flavell, R. A.; Fikrig, E. ASC/PYCARD and Caspase-1 Regulate the IL-18/IFN-γ Axis during Anaplasma Phagocytophilum Infection. *The Journal of Immunology* **2007**, *179* (7), 4783–4791. https://doi.org/10.4049/jimmunol.179.7.4783.

(11) Brainard, A. M.; Korovkina, V. P.; England, S. K. Disruption of the Maxi-K-Caveolin-1 Interaction Alters Current Expression in Human Myometrial Cells. *Reproductive Biology and Endocrinology* **2009**, *7* (1), 131. https://doi.org/10.1186/1477-7827-7-131.

(12) Smith, T. K.; Hager, H. A.; Francis, R.; Kilkenny, D. M.; Lo, C. W.; Bader, D. M. Bves Directly Interacts with GEFT, and Controls Cell Shape and Movement through Regulation of Rac1/Cdc42 Activity. *Proceedings of the National Academy of Sciences* **2008**, *105* (24), 8298–8303. https://doi.org/10.1073/pnas.0802345105.

(13) Bernatchez, P. N.; Sharma, A.; Kodaman, P.; Sessa, W. C. Myoferlin Is Critical for Endocytosis in Endothelial Cells. *American Journal of Physiology-Cell Physiology* **2009**, *297* (3), C484–C492. https://doi.org/10.1152/ajpcell.00498.2008.

(14) Wang, C.; Ruan, R.; Zhang, L.; Zhang, Y.; Zhou, W.; Lin, J.; Ding, W.; Wen, L. Role of the Na/K -ATPase Beta-Subunit in Peptide-Mediated Transdermal Drug Delivery. *Mol Pharm* **2015**, *12* (4), 1259–1267. https://doi.org/10.1021/mp500789h.

(15) Nonami, A.; Taketomi, T.; Kimura, A.; Saeki, K.; Takaki, H.; Sanada, T.; Taniguchi, K.; Harada, M.; Kato, R.; Yoshimura, A. The Sprouty-related Protein, Spred-1, Localizes in a Lipid Raft/Caveola and Inhibits ERK Activation in Collaboration with Caveolin-1. *Genes to Cells* **2005**, *10* (9), 887–895. https://doi.org/10.1111/j.1365-2443.2005.00886.x.
